# Estimating parameters of nonlinear dynamic systems in pharmacology using chaos synchronization and grid search

**DOI:** 10.1007/s10928-019-09629-4

**Published:** 2019-03-30

**Authors:** Nikhil Pillai, Sorell L. Schwartz, Thang Ho, Aris Dokoumetzidis, Robert Bies, Immanuel Freedman

**Affiliations:** 1 Computational and Data-Enabled Science, University at Buffalo, Buffalo, USA; 2 Department of Pharmacology and Physiology, Georgetown University Medical Center, Georgetown, USA; 3 Vertex Pharmaceuticals, Boston, USA; 4 Department of Pharmaceutical Technology, University of Athens, Athens, Greece; 5 Pharmaceutical Science, University at Buffalo, Buffalo, USA; 6 Freedman Patent, Harleysville, PA 19438 USA

**Keywords:** Chaos synchronization, Parameter estimation, Chaotic system, Delay differential equation, Least squares

## Abstract

Bridging fundamental approaches to model optimization for pharmacometricians, systems pharmacologists and statisticians is a critical issue. These fields rely primarily on Maximum Likelihood and Extended Least Squares metrics with iterative estimation of parameters. Our research combines adaptive chaos synchronization and grid search to estimate physiological and pharmacological systems with emergent properties by exploring deterministic methods that are more appropriate and have potentially superior performance than classical numerical approaches, which minimize the sum of squares or maximize the likelihood. We illustrate these issues with an established model of cortisol in human with nonlinear dynamics. The model describes cortisol kinetics over time, including its chaotic oscillations, by a delay differential equation. We demonstrate that chaos synchronization helps to avoid the tendency of the gradient-based optimization algorithms to end up in a local minimum. The subsequent analysis illustrates that the hybrid adaptive chaos synchronization for estimation of linear parameters with coarse-to-fine grid search for optimal values of non-linear parameters can be applied iteratively to accurately estimate parameters and effectively track trajectories for a wide class of noisy chaotic systems.

**Electronic supplementary material:**

The online version of this article (10.1007/s10928-019-09629-4) contains supplementary material, which is available to authorized users.

## Introduction

Pharmacotherapy assumes a reasonable degree of predictability. However, the human body is a complicated dynamic system in which a large number of variables are in operation simultaneously.

J. M. van Rossum and J. E. C. M. de Bie, 1991 [1]

Learning and confirming are quite distinct activities, implying different goals, study designs, and analysis modes.

L. B. Sheiner, 1997 [2]

As noted elsewhere [3], the objectives of implementing mathematical models—with or without simulation—to explore the nature of biological systems span a continuum. It begins at the far left where the sole objective is to predict parameter values unambiguously and ends at the far right where the purpose is a biological ‘Theory of Everything,’ a fully mechanistic understanding of all systems and their interactions. While our modeling and simulation capabilities fall significant distances from either end of that continuum, it is necessary to know where we are and why we are there.

The quoted epigraphs are a fitting reminder to assess what can be achieved or expected with any particular approach to modeling or modeling and simulation.

Developments in pharmacokinetic (PK) and pharmacodynamic (PD) modeling, for all the vast leaps in progress made possible by the concomitant expansion of computational resources, still hover closer to the left (prediction) end of that continuum. Modeling and simulation of physiologically based pharmacokinetic–pharmacodynamics (PB–PKPD) allow for a myriad of *what if* questions and answers of critical importance to drug development. Nonetheless, the treatment of PB–PKPD from the biological systems and especially the disease systems (DS), perspective is of much more recent vintage. A major portion of this effort is applied to create networks describing PB–PKPD–DS interactions and recognize their complex dynamics. Danhof [4] classified dynamical systems into “non-adaptive” and “adaptive”—the former having properties that are functionally constant over time, the latter whose system properties may change, particularly through emergence of self-organization. Danhof also suggested an organization of “fundamental properties of therapeutic interventions on biological systems behavior,” viz. non-linearity, individuality, variability, interdependency, convergence, resilience, and multistationarity.

Multistationarity is a property of biological systems that may exist in multiple stable states. It is a property that may not be immediately apparent from modeling and simulation based on ordinary differential equations (ODEs). Bakshi et al. [5] presented an interesting demonstration of unexpected behavior in an ODE model of a nonlinear prolactin precursor pool model with feedback to account for the activity of antipsychotic drugs. Through a sophisticated dynamical systems analysis, they concluded that the model nonlinearity resulted in multistationarity, viz. two steady-states with differing stability levels. This multistationarity confounded their simulation, but the cause could not be immediately identified by visual inspection.

The epigraph by van Rossum and de Bie in 1991 introduced their proffer for considering the role of chaotic systems in understanding and predicting pharmacological response. The issues they raised included the implication that a shortcoming of pharmacodynamic modeling was a paucity of dynamics, as represented by empirical receptor occupancy models. Ever since, modeling pharmacodynamics has been addressed by sub-models varying in complexity that link time and concentration to concentration-effect; or more succinctly, a dynamic sub-model linking time and concentration with a non-linear static one linking concentration to effect. In suggesting the possibility that a chaotic process could underlie pharmacological mechanisms, van Rossum and de Bie put forward the involvement of nonlinear dynamic systems, specifically chaotic systems. The origin of their proffer was, in good part, teleological. The emerging literature, particularly in EEG and ECG analysis indicated the possibility of chaotic functions that accommodated positive and negative feedback control; which they saw as applicable to the complexity of pharmacodynamic actions.

Dokoumetzidis et al. [6] concisely reviewed the basic principles of nonlinear dynamic systems and chaos and considered how they can be applied to PD systems. A classical and relatively uncomplicated presentation of mathematical chaos theory is the logistic map based on the following difference equation for population growth

1 $$X_{{{\text{n}} + 1}} = rX_{\text{n}} (1 - X_{\text{n}} )$$

where 1 > *X *> 0.

X is a variable which defines the state of a dynamical system and r is a constant.

Up to *r *≈ 2.5, a plot of *X*_n_ versus iteration number will settle at a single value $\frac{r - 1}{r}$(attractor), irrespective of the starting value of *X*. As r increases above 2.5, the map will undergo a periodic doubling, but coming to the same attractor values independent of starting value. Somewhere between *r* = 3.5 and 4 (depending on the computer’s floating point calculations), the map will appear almost random, and the points on the map will be starting point dependent. A return map plot of *X*_n_ versus *X*_n+1_ will reveal a non-random distribution.

**Fig. 1:**
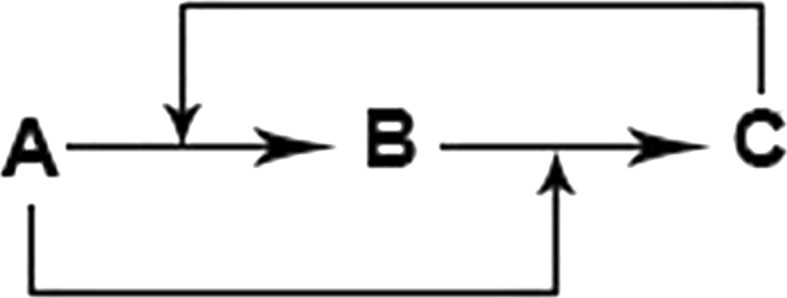
Schematic of Belousov–Zhabotinsky (B–Z) reaction showing information exchange between different components

Gontar [7] demonstrated the usefulness of difference equations in modeling the Belousov–Zhabotinsky (B–Z) reaction as an analogy illustrating forward and backward control mechanisms. The B–Z reaction is a classic example of a non-linear chemical oscillator from a non-equilibrium state that will, depending on the reactants, colorfully demonstrate the oscillations. There are multiple steps to the reactions, but Gontar was able to mathematically describe, via difference equations, the empirical reaction data from the B–Z reaction in the form of a model illustrated by the diagram in Fig. 1.

Figure 1 shows the information exchange wherein the concentration of component C affects the reaction rate of A to B and the concentration of component A affects the reaction rate of B to C. It is not difficult to see a corresponding representation for modeling the complex dynamics of living systems. In the case of pharmacodynamics, it could represent systems more complex than generated by receptor occupancy mechanisms without feedback and by presumptions of commonly accepted linear and non-linear pharmacokinetic factors affecting local target site concentration.

However, if the system is chaotic, that modeling is problematic. As noted, chaotic systems are starting value dependent and as illustrated using the foregoing logistic equations, miniscule changes in the controlling functions (as with *r* above) can have a significant effect on the outcome although the outcomes can display long-range order and cyclic patterns of outcomes can recur closely from time to time. Thus, the commonly implemented stochastic approaches characterizing and predicting system behavior are likely to be inadequate for prediction in the time domain.

There are numerous examples of nonlinear dynamical systems in pharmacodynamics. Pharmacodynamics traditionally has been based on a receptor occupation theory without feedback. This consideration leads to the classical direct and indirect Emax response models which are often applied in PD studies. However, deviations from this behavior can be anticipated when an endogenous substance e.g., a hormone or a neurotransmitter, is considered and a feedback mechanism, induced by the formation of a ligand–receptor complex, operates to maintain a basal ligand value. Indeed, Tallarida [8] has analyzed such a system using techniques of nonlinear dynamics and has shown that this system can be either dynamically stable or unstable, depending on the values of the parameters involved. These theoretical results were confirmed experimentally in a quantitative study of the control of dopamine release by negative feedback in the rat striatum [9].

It is widely appreciated that hormone secretion is characterized by pulsatility. The first experimental studies of the pulsatile nature of hormone secretion started more than 30 years ago. Hellman et al. reported in 1970 [10] that “Cortisol is secreted episodically by normal man.” It was also realized that this pulsatility was not due to noise but was actually associated with physiological processes. Indeed, the circadian clock, the interaction between hormones through feedback mechanisms, and the interaction of hormones with central and autonomic nervous systems are some of the reasons for this behavior. It has been apparent that the theory of dynamical systems is the right field to find useful tools for the study of hormonal systems. Kuznetsov et al. [11] presented a mathematical model of the cytotoxic T lymphocyte response to the growth of an immunogenic tumor. As stated by Kuznetsov”, The model exhibits a number of phenomena that are seen in vivo, including immunostimulation of tumor growth, ‘sneaking through’ of the tumor, and formation of a tumor ‘dormant state’”. By comparing the model with experimental data, numerical estimates of parameters describing processes that cannot be measured in vivo were also derived. He said that there are different explanations for the termination of a tumor dormant state, for sneaking through of tumors, and for immunostimulation effects. Often these explanations are based on the ideas of immunoselection, antigenic modulation, production by tumor cells of different types of immune cell blocking factors, generation of immunosuppressor cells, changes in auto-regulatory networks in a tumor localization region, and other more complex ideas that are challenging to prove or disprove experimentally. He proposed that these phenomena may be the result of nonlinear dynamic interactions between the tumor and the immune system.

Classical approaches for tracking and determining parameter estimates of these nonlinear chaotic systems are very unreliable and Konnur [12] stated the key issues while estimating parameters of these dynamical systems. First, the method needs to be robust in the presence of noise. Second, the method must allow estimation of all parameters using any conveniently measurable output from the system. Third, it must be able to rapidly track changes in the operating parameters of the experimental system. For a system with multiple parameters, the least squares objective function possesses multiple minima [12] and this can lead to an erroneous estimate of parameters owing to convergence at one of the local minima. Also, for some cases, this method is not able to accurately track changes in operating parameters. The error in all these approaches increases with an increase in noise and decrease in data density.

Since the solutions of a chaotic system may be multi-modal and non-convex, key challenges [33] include (1) very flat objective function surface in the vicinity of the solution; (2) over-determined models; (3) badly scaled model functions and, (4) non-differentiable terms with respect to systems dynamics. Since classical methods have low accuracy and are computationally expensive, one can look for methods which are robust and can overcome these issues. Controlled chaos synchronization is one approach that may be appropriate to address these problems [13].

## Methods

### Model used in current study

**Fig. 2:**
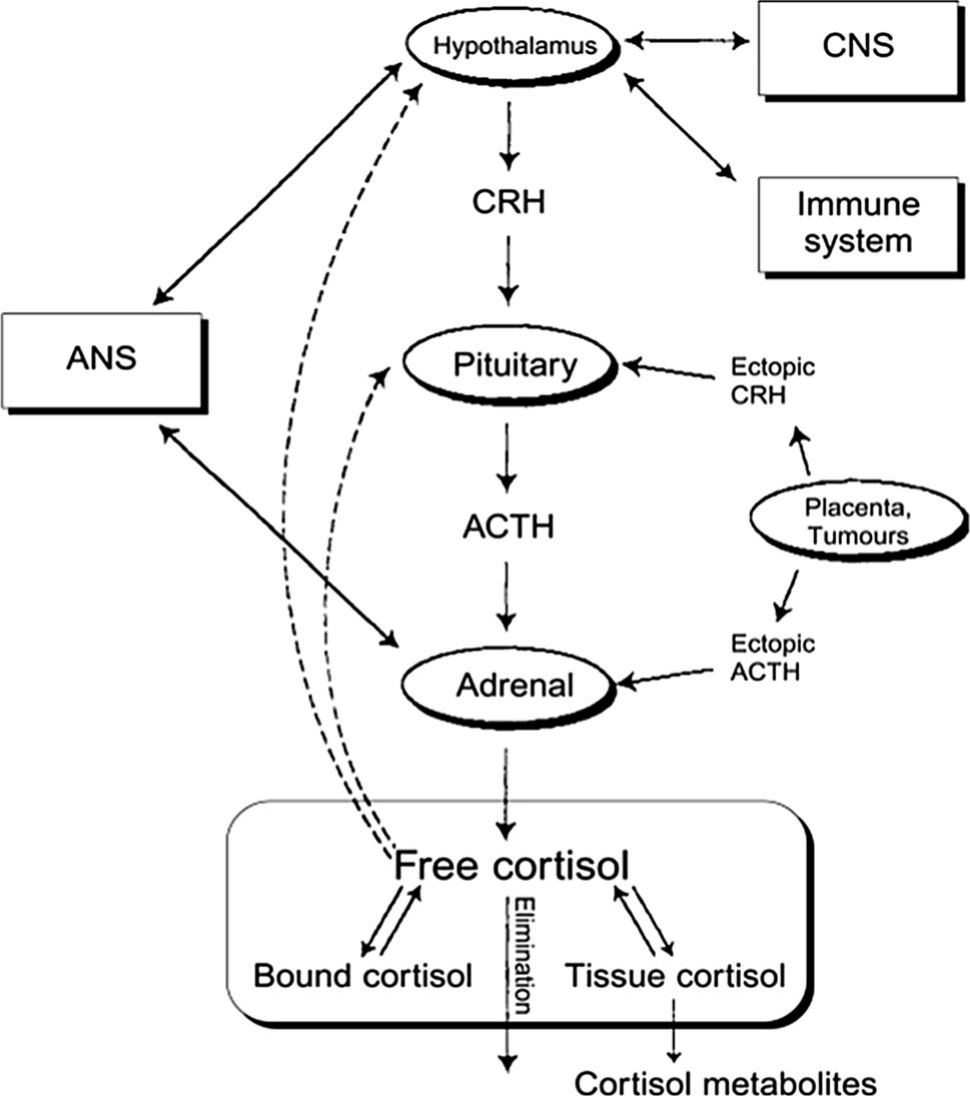
A schematic representation of the hypothalamic–pituitary–adrenal axis (HPA), together with other organs and systems that interplay in the secretion of cortisol. Solid arrows indicate stimulation, production or reaction and dashed arrows inhibition, while double arrows indicate more complicated bi-directional interaction. At the bottom of the graph the various components of cortisol disposition are indicated. Free cortisol, which participates in the feedback mechanism, is also in equilibrium with the cortisol species bound to corticosteroid bound globulin and tissue cortisol. In experimental studies, the measured blood cortisol levels are the sum of free and bound cortisol. Key: ANS (autonomic nervous system), CNS (central nervous system) (after [15])

The hypothalamic–pituitary–adrenal axis (HPA) is one of the most widely studied hormonal systems (see Fig. 2). The model is nonlinear. In this model, cortisol concentration is described by a nonlinear time-delay differential equation [14, 15] with two terms, a secretion rate term which adheres to a negative feedback mechanism [16, 17] and drives the pulsatile secretion and, a first order output term.

2 $$\frac{dC\left( t \right)}{dt} = \frac{{k_{1} a^{n} C\left( {t - d} \right)}}{{a^{n} + C\left( {t - d} \right)^{n} }} - k_{2} C\left( t \right)$$

where $a = \alpha \cos \left( {\left( {t - t_{f} } \right)\frac{2\pi }{1440}} \right) + \beta$ and C is the cortisol concentration, C_d_ (C(t − d)) is the value of C at time (t − d), n is the exponent which controls the oscillations in the cortisol model, *k*_*1*_ is the input rate constant and *k*_*2*_ is the output rate constant. The complex disposition characteristics are modeled with a simple first-order rate constant, *k*_*2*_. The circadian rhythm of cortisol secretion is implemented by considering the ‘$a$’ parameter of the model as a simple cosine function of the 24 h circadian period where α and β are constants with concentration units, t_f_ is a constant with time units and t is the time in minutes [18]. The nominal parameter values are listed in Table 1.

**Table 1:** Parameter list with nominal values for the cortisol model in [15] together with initial values and property

Parameter	Nominal value	Property	Initial value
Input rate constant, *k*_*1*_ (1/min)	0.0666	Linear	1
Output rate constant, *k*_*2*_ (1/min)	0.0333	Linear	0.01
Constant, α (μg/100 ml)	0.7	Nonlinear	0.01
Constant, β (μg/100 ml)	1	Nonlinear	0.1
Phase, t_f_ (min)	250	Nonlinear	0.1
Delay, d (min)	70	Nonlinear	50
Fixed switch exponent, n	10	Nonlinear	Fixed

Parameters that enter in a linear fashion were estimated using adaptive chaos synchronization while the parameters that enter in a nonlinear fashion were estimated by combining grid search with adaptive chaos synchronization

### Data generation and processing

**Fig. 3:**
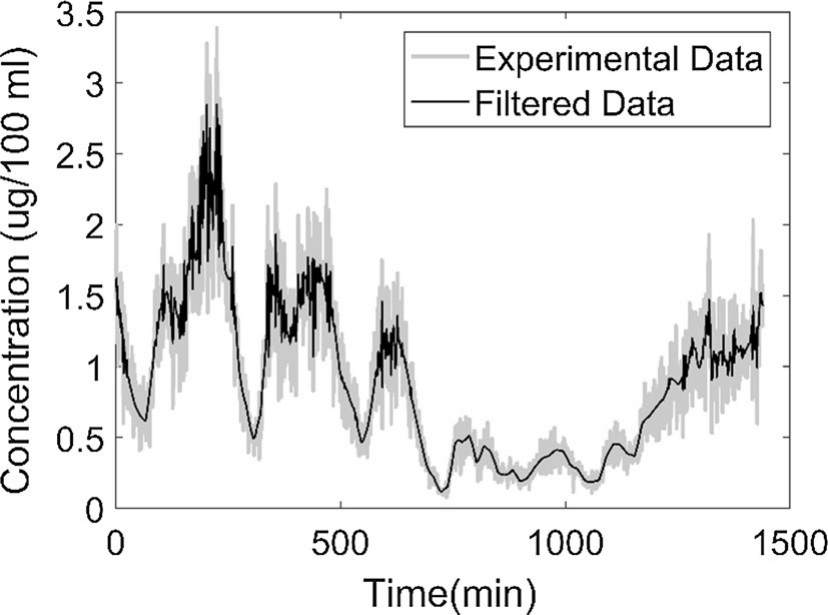
The experimental data included undesired information in the form of noise. To estimate parameters given these noisy data we need to filter the undesired information without losing too much desired information. The data were filtered by wavelet denoising according to a **wden** function in MATLAB. The filtered data are input to the chaos synchronization estimation method based on least squares or extended least squares objective functions

Four datasets were considered to demonstrate the utility of the chaos synchronization function. Thus, the algorithm was applied to a noiseless dataset, noisy datasets (with 20% and 50% proportional noise) and a noiseless sparse dataset. The simulated experimental ODE system was generated by solving Eq. (2) using the **dde23** function in MATLAB 2017a, which solves delay differential equations with constant delays. We validated the trajectory generated by the solver by comparing with the results provided in [18]. The value of the history function was fixed to 1.7, which is the initial value of cortisol concentration. To generate a noisy system, the simulated experimental noisy data were generated from a normal distribution with mean equal to the concentration value of the experimental system and 20% proportional error using the **randn** function in MATLAB(Mathworks:MA) version 2017a (see Fig. 3).

Data processing and information extraction from the above datasets required noise filtering as an initial step. We used the **wden** function with a ‘symmlet’ basis expanded to level 4 in MATLAB 2017a. This function performs an automatic de-noising process of a one-dimensional signal by thresholding wavelet components. The input options to the function can be changed according to the noise level. This function was found to be more effective for noisy chaotic systems than a moving average filter (**filter** function) because it preserves geometric information of the trajectory. Parameter estimation and system tracking were performed on this filtered signal. We found that, unless we preserve the geometric information, nonlinear parameters could not be accurately estimated. While estimating the parameters using grid search, filtering was performed at level 4 and when the parameters were being estimated using nonlinear least squares, extended least squares or chaos synchronization, the filtering was performed at level 1. It was observed that chaos synchronization method performs more accurately with a lower level of filtering that preserves more detail. As stated in [19], noise can induce the synchronization by stochastic resonance.

Let us now consider various approaches for estimating parameters of the cortisol system.

### Non-linear least squares regression

Classical approaches such as least squares regression and maximum likelihood are widely used for tracking non-linear systems. Many of these classical approaches are numerical and, to achieve synchronization, they require the leading Lyapunov exponent to be negative. However, it has been recently reported that local (rather than global) negativity of conditional Lyapunov exponent is neither a necessary nor a sufficient condition to guarantee chaos synchronization [20, 21].

In the method of nonlinear least squares, we pick the coefficients β to minimize the residual sum of squares

3 $$RSS\left(\upbeta \right) \approx \mathop \sum \limits_{i = 1}^{N} \left( {y_{i} - \varSigma_{J} J_{ij} \beta_{j} } \right)^{2}$$

where $y_{i}$ is the observed value,$J$ is the Jacobian, $J_{ij} \beta_{j}$ is the fitted value and RSS(β) is a quadratic function of parameters whose minimum always exists, but may not be unique [22].

We fitted the Dokoumetizidis cortisol model by nonlinear least squares using the **lsqcurvefit** function in MATLAB version 2017a to fit linear parameters and grid search to fit nonlinear parameters. This function requires the x data i.e. time of observation, y data i.e. observed concentration, model function and initial values for all the parameters to be estimated. The initial values of all the parameters were set reasonably close to their nominal values (Table 1).

### Extended least squares

In the case of Extended Least Squares (ELS) the variance of error becomes a function of the predicted values at each time point, rather than a function of the corresponding data values. The objective value function for ELS is given by:

4 $$obj_{ELS} \left( {\theta , \sigma } \right) = \mathop \sum \limits_{n = 1}^{n} \frac{{\left( {C_{i} - M\left( {\theta ,t_{i} } \right)} \right)^{2} }}{{V\left( {\theta ,\sigma ,t_{i} } \right)}} + \ln V\left( {\theta ,\sigma ,t_{i} } \right)$$

where $V\left( {\theta ,\sigma ,t_{i} } \right)$ is a variance model, $C_{i}$ represents the observed data, $M\left( {\theta ,t_{i} } \right)$ represents the predicted data, $\theta$ denotes the vector of parameters of the model, and $\sigma$ denotes the standard deviation [23]. In the presence of noise, ELS performs better than Root Mean Square Error (RMSE) due to the weighting scheme implemented in ELS. This is important for nonlinear dynamic systems since they may possess multiple local minima.

### Chaos synchronization

**Fig. 4:**
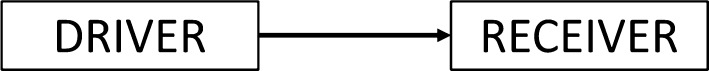
A schematic representation of a unidirectional coupling configuration. The solid arrow indicates the direction of flow of information

Chaotic systems are dynamic systems which are highly sensitive to initial conditions. As a result, two identical chaotic systems starting with nearly the same initial conditions can have exponential separation of trajectories with time [24]. Chaos synchronization refers to a controlled synchronization process wherein two or more chaotic systems adjust a given property (either equivalent or non-equivalent) of their motion to a common behavior due to a coupling or forcing [24]. This common behavior ranges from a complete agreement of trajectories to locking of phases. Based on the coupling configuration there is a great difference in the processes leading to synchronized states. There are two types of coupling configurations; the first is unidirectional coupling and the second is bidirectional coupling. Unidirectional coupling entails the global system being realized by two subsystems with a drive-response configuration (see Fig. 4). One subsystem (driver) evolves freely and drives the evolution of the other. The response system is “slaved” to follow the dynamics of the driver system; examples of such systems are cortisol secretion and immune-tumor cell interactions. On the other hand, for a bidirectional system, both subsystems are coupled with each other. These types of systems are more complicated to work with.

The coupling factor induces adjustment of cyclic rhythms onto a common synchronized manifold resulting in mutual synchronization behavior. Examples of such systems are physiological systems such as cardiac, neural and respiratory systems which are bi-directionally coupled. When we talk about coupled identical systems, synchronization appears as near equality of a state variable evolving in time. This type of synchronization is referred to as complete synchronization. The existence of complete synchronization implies that the response system is asymptotically stable i.e. the difference between the outputs of the driver and slave systems asymptotically tends to zero. For complete synchronization, there is asymptotically perfect locking of chaotic trajectories of two systems achieved by means of a coupling signal and the trajectories evolve to remain in step with each other in the course of time [24].

In this paper, we concentrate on an adaptive coupling scheme proposed by Huang [25] which supplies a simple, analytical and systematic controller to synchronize almost arbitrary similar chaotic systems that are uniform Lipschitz, including systems whose first derivatives (or Jacobian) are of bounded variation everywhere.

He proved by using the invariance principle [26] of differential equations that a linear feedback coupling with an adaptively-updated feedback strength proportional to the square of the residuals can asymptotically perfectly synchronize two almost arbitrary identical chaotic systems [25]. This method can solve for all parameters which appear in a linear fashion and the asymptotic global stability guarantees asymptotic global exponential stability in the noiseless case, i.e. asymptotic global negativity of conditional Lyapunov exponents for the augment system (receiver).

In mathematics, a Lyapunov exponent of a dynamical system is a quantity that characterizes the rate of separation of infinitesimally close trajectories [27]. Quantitatively, two trajectories in phase space with separation $\delta {\text{Z}}_{0}$ diverge at a rate given by

5 $$\left| {\delta {\text{Z}}({\text{t}})} \right| \approx {\text{e}}^{{\uplambda{\text{t}}}} \left| {\delta {\text{Z}}_{0} } \right|$$

6 $$\uplambda = \mathop {\lim }\limits_{t \to \infty } \mathop {\lim }\limits_{{\delta {\text{Z}}_{0} \to 0}} \frac{1}{t}\ln \frac{{\left| {\delta {\text{Z}}\left( t \right)} \right|}}{{\left| {\delta {\text{Z}}_{0} } \right|}}$$

where λ is the leading Lyapunov exponent.

By inspection of Eq. (6), the leading Lyapunov exponent is inversely proportional to the time to convergence.

To use this approach, the model structure must be already known, including the number of independent variables and the structure of the underlying equations. The augment system is based on artificial unidirectional coupling to a replica of the system. We apply the approach to the cortisol secretion model developed by Dokoumetzidis et al. [18].

We synchronized the system by unidirectional (drive-response) coupling (see Fig. 4). This coupling is realized by two subsystems, one of which evolves freely (the Driver) and drives the evolution of the other (the Response/Receiver).

Within this coupling configuration, the robustness of synchronization depends on the coupling scheme. Our research is based on the coupling scheme proposed by Huang [25] which guarantees an asymptotically non-positive global conditional Lyapunov exponent for the sequence of residuals in the noiseless case [28] and is discussed in the following section.

#### Huang’s adaptive coupling scheme

We applied adaptive chaos synchronization according to Huang’s scheme to the cortisol model [18], for which the chaotic system (driver System) is given by Eq. (2) and the time series for the concentration of cortisol C(t) is available as the experimental output of the cortisol system. We note that synchronization does not always require all the variables to be available, especially when the system is represented by a time-delay embedding [35]).

To estimate all unknown linear parameters from these time series we defined a receiver system whose governing equations are identical to the driver system although corresponding parameters may not necessarily take the same values. The evolution of the receiver system is guided by the driver trajectory by means of the driving signal (first and second terms of Eq. 7). These parameters are initialized to arbitrary initial values and their values are updated iteratively until complete synchronization is obtained.

The receiver system is controlled by adaptive linear feedback according to the following equation

7 $$\frac{dC\left(t \right)}{dt} = \frac{{k_{1} a^{n} C\left({t - d} \right)}}{{a^{n} + C\left({t - d} \right)^{n}}} - k_{2} C\left(t \right) + \epsilon \left(t \right) \times \left({C\left(t \right) - C_{obs} \left(t \right)} \right)$$

where $\left( {C\left( t \right) - C_{obs} \left( t \right)} \right)$ is the synchronization error denoted by $e_{i}$ and $\epsilon(t)$ is the time-dependent feedback strength.

8 $$e_{i} = \left( {C\left( t \right) - C_{obs} \left( t \right)} \right)$$

The feedback strength $\epsilon_{i}$ is then adapted according to the following nonlinear update law

9 $$\frac{d\epsilon(t)}{dt} = - \varGamma \times \left({e_{i}} \right)^{2}$$

where $\varGamma$ is a tuning parameter that affects the rate of convergence.

The parameters are adapted according to the following update rules which are similar to the delta rules encountered in machine learning

10 $$\frac{{dk_{1} \left( t \right)}}{dt} = - \delta_{1} \times \left( {e_{i} } \right) \times dF_{1}$$

where $$dF_{1} = \frac{{a^{n} C\left( {t - d} \right)}}{{a^{n} + C\left( {t - d} \right)^{n} }}$$

11 $$\frac{{dk_{2} \left( t \right)}}{dt} = - \delta_{2} \times \left( {e_{i} } \right) \times dF_{2}$$

where $$dF_{2} = - C\left( t \right)$$.

Here $\delta_{1}$ and $\delta_{2}$ are tuning parameters that also affect the rate of convergence. The values of $\delta_{1} = \delta_{2 } = 1$ and $\varGamma = 1$ were utilized and fixed based upon the conclusion derived from the Lyapunov exponent and tuning parameter analysis presented in the Results section of this paper.

Theoretically, the receiver system should track the driver system accurately for any value of $\varGamma$ and $\delta$, however, the key issue is the rate of convergence of parameters, especially for noisy data. The values of $\varGamma$ and $\delta$ were chosen so that even for noisy systems the parameters converge to about their nominal value (given in [18]) within about 6 cortisol cycles (about 6 days) of the sparsely sampled time series.

These equations were solved in MATLAB using the **dde23** solver with initial values (*k*_*1*_= 0.01, *k*_*2*_= 0.01 $\epsilon = 0.1$) and an initial concentration of cortisol set to 1.7 μg/100 ml.

Although the adaptive chaos synchronization method identifies the parameters that appear in a linear fashion it is not immediately applicable to parameters that enter in a nonlinear fashion. To estimate these parameters, we combined grid search with chaos synchronization.

When CS estimates parameters that enter into a noiseless system of uniform Lipshitz ODEs in a linear fashion, it converges to a global minimum because the Lasalle Invariance Principle of ODEs applied to trajectories of the augmented system consistent with a Lyapunov function that is constructed to be monotonic decreasing guarantees that the sequence of residuals is asymptotically stable at the origin, see Huang [29]. A denoised system or a system with only a few percent of noise still converges to the global minimum with intermittent fluctuations due to phase slips that can be resolved by filtering or averaging.

Theesar et al. [35] extends the Huang [29] analysis to uniform Lipshitz Stochastic Differential Equations (SDEs) with Wiener noise, with similar results based on the Lasalle Principle for SDEs i.e., on the trajectories of their augmented system, the sequence of residuals is asymptotically stable at the origin. To understand how the nonlinear parameters may be estimated, please note that dissimilar systems may be synchronized using trajectories of an augmented system according to a Lyapunov function that has been constructed without updating i.e., the learning rate is set to zero. Since dissimilar systems with different parameter sets can be synchronized in the sense that, when constrained to trajectories of their augmented system the sequence of residuals is asymptotically stable at the origin, the search method can search for linear or nonlinear parameters with the guarantee that the sequence of residuals will remain bounded and cannot spiral outwards from the origin on trajectories of the augmented system.

### Grid search

A traditional approach to hyperparameter optimization has been grid search or parameter sweep, which is simply an exhaustive search through a specified subset of the hyperparameter space of a learning algorithm. A grid search algorithm must be guided by some performance metric, which we selected to be the RMSE between the observed and predicted concentrations.

Grid search is particularly useful because we cannot guarantee the existence of any derivatives higher than the first nor can we guarantee the local continuity of the objective function in the time domain for the uniform Lipschitz systems considered by Huang [29]. While the system remains on the trajectories of the augment system, this confers global asymptotic stability according to the invariance principle provided the largest invariant set of residuals to which the trajectories converge, converge to a unique value zero. If the sequence of residuals were asymptotically not chaotic, grid search would be expected to be as effective as for the parameters of a similar system that was not in a chaotic regime.

#### How grid search works

We created a grid for the variables α and β in the cortisol model [Eq. (2)]. The grid range was selected based on multiple runs. After each run, the lower and upper limits of the grid were modified, and the grid was refined to obtain precise estimates of parameter values.

We parse the entire grid by nested loops and for each combination of parameter (α and β) values obtained from the loop, call the chaos synchronization function, estimate the values of *k*_*1*_ and *k*_*2*_ by chaos synchronization and determine the cortisol concentration at output time points. We compare the predicted concentration with the observed concentration and store the Root Mean Square Error (RMSE).

When the complete grid has been parsed, we store the values of parameters corresponding to the minimum value of RMSE.

### Sensitivity analysis

We evaluated the sensitivity of the predicted cortisol concentration to various parameter values to determine the uncertainty in the model output. Sensitivity analysis is defined as the study of how uncertainty in the output of a model can be attributed to different sources of uncertainty in the model input [30].

Each parameter was varied according to a pre-specified grid defined in Table 2. While the parameter under consideration was varied, all other parameters were set to their nominal values in Table 1.

**Table 2:** Mesh for sensitivity analysis

Parameter	Initial value	Final value	Step size
n	1	20	1
*k*_*1*_	0.01	0.1	0.01
*k*_*2*_	0.01	0.1	0.01
α	0.1	2	0.1
β	0.1	2	0.1
t_f_	50	700	50

The RMSE was calculated by comparing the concentration obtained for the nominal parameter values and the concentration obtained with the new set of parameters. We can visualize the sensitivity by inspecting graphs of total error versus parameter value.

### Error metric

RMSE between the observed and predicted concentration was employed as a metric to measure the error and to find the optimum configuration from the grid search algorithm.

When we fitted data using chaos synchronization we observed transient effects during a burn-in period. To reduce the impact of transient effects we implemented oversampling. In this approach, we generated sample sets longer than needed and early samples were discarded.

In the dense data and noisy data cases, we sampled data equivalent to about two cortisol cycles and discarded output from the first cycle. The error was calculated by comparing the data observed and predicted in the second cortisol cycle. For the sparsely sampled case, we sampled the data equivalent to about six cortisol cycles and discarded the first five cortisol cycles. The error was calculated by comparing the data observed and predicted in the sixth cortisol cycle. For the sparsely sampled case, due to the density of the data, grid search required an increased number of data points to accurately estimate the optimum parameter values. To compare the accuracy with which the final parameters were estimated across configurations, we calculated the percent error.

## Results

### Pre-processing experimental data

We applied the **wden** function to filter noise from the data. Parameter estimation and system tracking were performed on this filtered signal (Fig. 3). For a fair comparison between the estimation methods, the same input options were selected for filtering the data.

### Estimating linear parameters

We initially compared the performance of the classical least squares method and extended least squares method with chaos synchronization while estimating only the linear parameters, *k*_*1*_ and *k*_*2*_ with initial parameter values of *k*_*1*_ and *k*_*2*_ set to 0.01. The nonlinear parameters were fixed to their nominal value.

We considered four scenarios with respect to data density and noise level. In the first scenario, we considered the dataset to be noiseless with data points at 1-min intervals. In the second and third scenario, a proportional noise of 20% or 50% was applied to the data set. In the fourth scenario, we considered a noiseless data set sampled at 45-min intervals. Tables 3 and 4 illustrate the results of the analysis when we estimated linear parameters using all three methods. It may be feasible to obtain clinical cortisol data sampled at 45-min intervals via microdialysis.

**Table 3:** The RMSE between the predicted and the observed concentrations for various methods of parameter estimation

Dataset	RMSE			
	NLS	CS/grid search	NLS/grid search	ELS/grid search
No noise, dense data^a^	0.89	2.76e−04	1.07	0.5013
20% proportional error, dense data^a^	0.93	0.0131	1.11	0.4973
50% proportional error, dense data^a^	1.03	0.0162	1.07	0.5241
No noise, sparse data^b^	0.91	0.0019	1.06	0.5029

The RMSE is measured over one cortisol cycle with units μg/100 ml

^a^1 min sampling interval

^b^45 min sampling interval. RMSE when only the parameters that enter in a linear fashion were estimated by adaptive chaos synchronization is the same as the RMSE obtained when all parameters were estimated using a combination of adaptive chaos synchronization and grid search

**Table 4:** Estimation of parameters that enter in a linear fashion by chaos synchronization (CS), nonlinear least squares (NLS) and extended least squares (ELS)

Estimation method	Nominal	CS	NLS	ELS
Noiseless system with dense data^a^				
*k*_*1*_	0.0666	0.069	0.01	0.0666
*k*_*2*_	0.0333	0.0344	0.01	0.0333
Noisy system^b^ with dense data^a^				
*k*_*1*_	0.0666	0.0629	0.01	0.0666
*k*_*2*_	0.0333	0.0340	0.01	0.0333
Noiseless system with sparse data^c^				
*k*_*1*_	0.0666	0.0615	0.01	0.0666
*k*_*2*_	0.0333	0.0303	0.01	0.0333

To estimate parameters for the noisy system we filter the data using the **wden** function provided by MATLAB (MathWorks: MA) version R2017a as input to the CS, NLS or ELS method

^a^Dense data signifies data sampled at 1 min intervals

^b^Noisy system signifies 20% proportional error

^c^Sparse data signifies data sampled at 45 min intervals

#### Estimating linear parameters using classical non-linear least squares regression

The cortisol model was solved using classical non-linear least squares regression with four scenarios with respect to data density and noise level. In all four scenarios (discussed in the above section) the least squares signal fails to track the data accurately. A substantial offset between the least squares signal and the observed values can be observed (see supplementary). Also, from Table 3 the error in system tracking using this classical approach is about 1000-fold higher when compared to system tracking using our proposed chaos synchronization approach for noiseless data (both dense and sparse) and, the error is about 70-fold higher for noisy data.

#### Estimating linear parameters using extended least squares

**Fig. 5:**
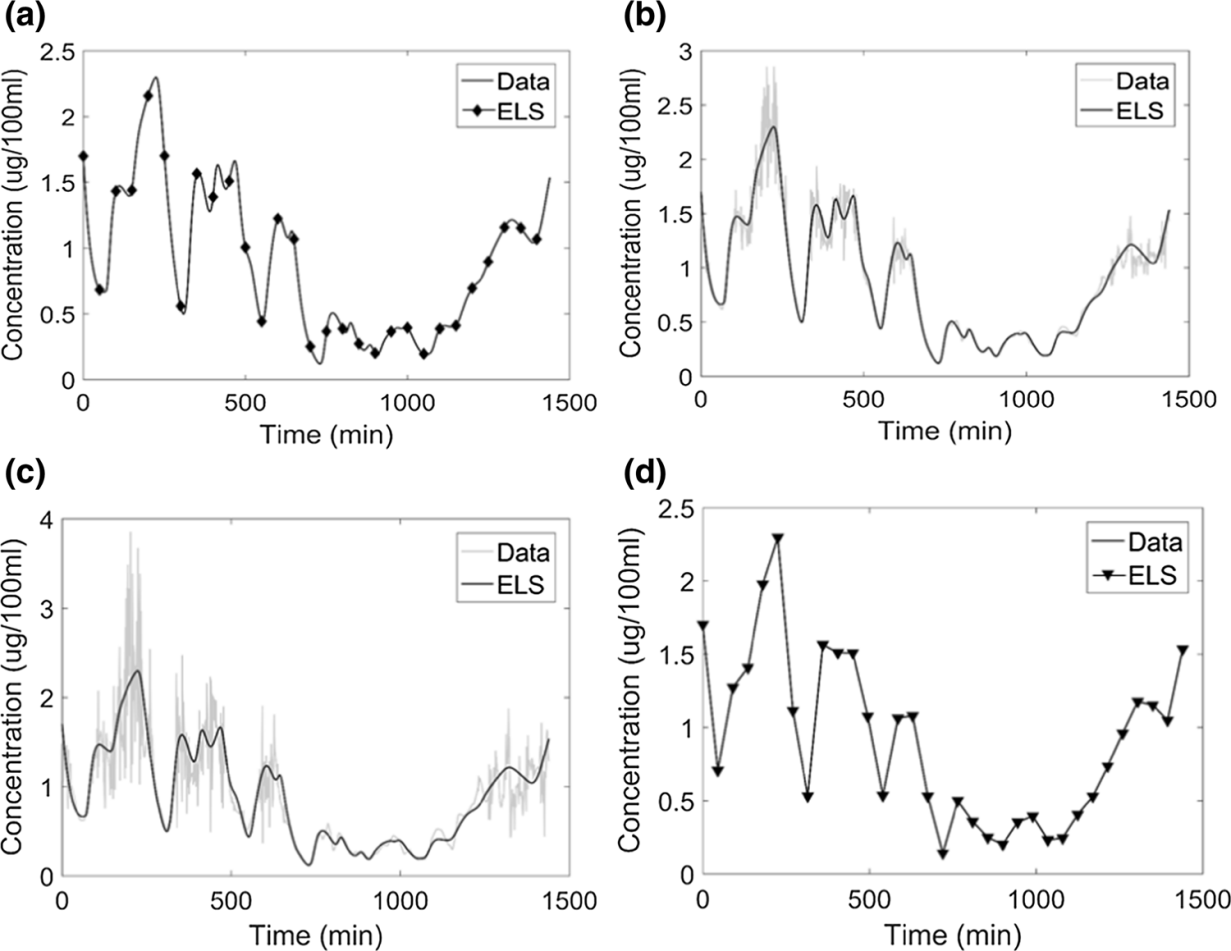
The system tracking performance of Extended Least Squares regression: **a** noiseless data sampled at 1 min intervals; **b** data with 20% proportional error sampled at 1 min intervals; **c** data with 50% proportional error sampled at 1 min intervals; **d** noiseless sparse data with data measured at 45 min intervals. The data were filtered by wavelet denoising. The line marked ELS represents the fit obtained using extended least squares regression. There is a small phase offset between case **a** and cases **b** or **c**

The cortisol model was solved using the ELS method with four scenarios with respect to data density and noise level. It was observed that when we were estimating linear parameters, the ELS method was found to be very accurate (Table 4). A slight offset can be observed for tracking of noisy data (panel b and c) in Fig. 5. In this case, the ELS objective function was used within a grid search to estimate linear parameters.

#### Estimating linear parameters using adaptive chaos synchronization

**Fig. 6:**
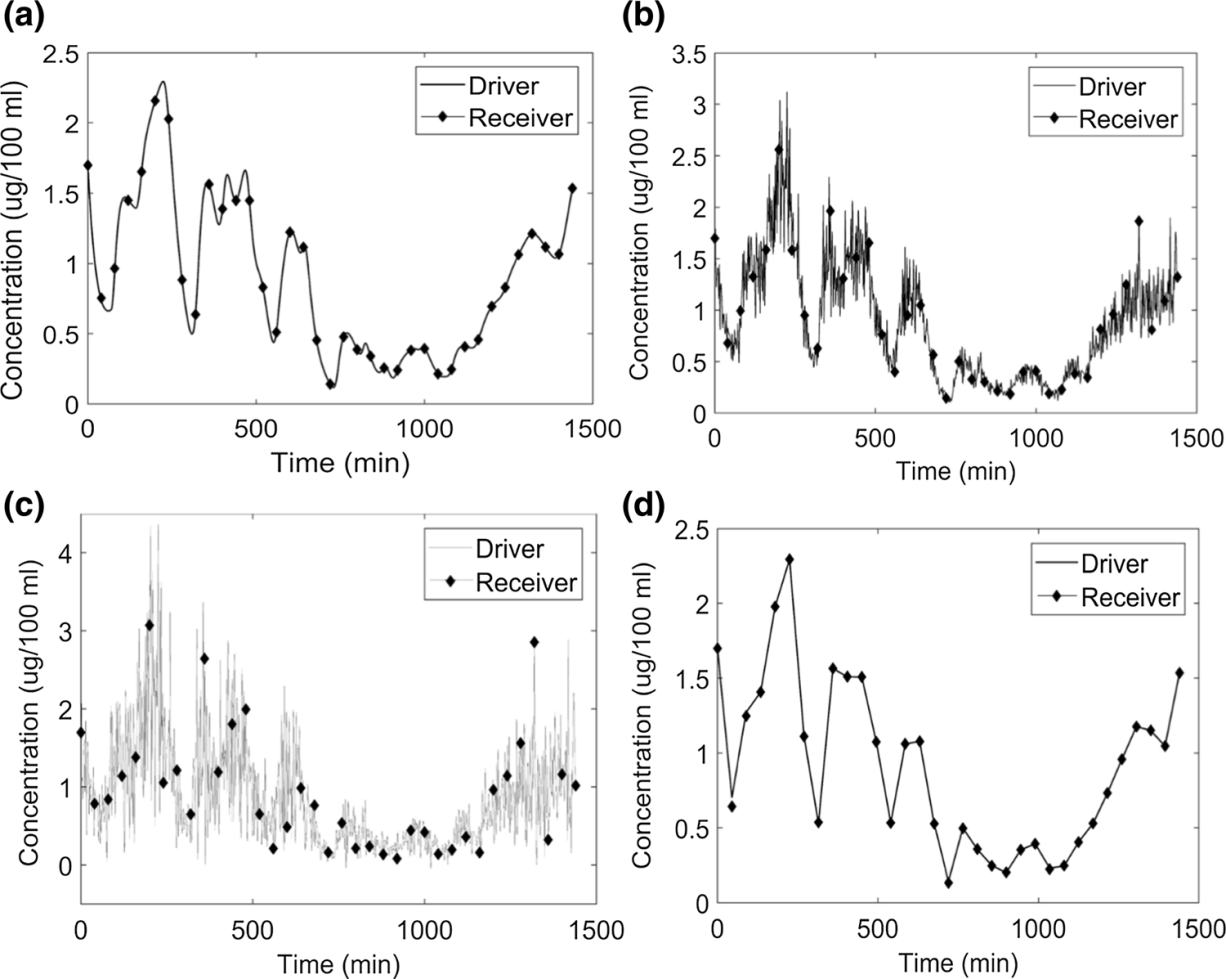
The system tracking performance of the adaptive chaos synchronization method: **a** noiseless data sampled at 1 min intervals **b** data with 20% proportional error sampled at 1 min intervals; **c** data with 50% proportional error sampled at 1 min intervals; **d** noiseless sparse data sampled at 45 min intervals. The data were filtered by wavelet denoising. Predictions based on chaos synchronization and grid search track the input data with relatively high accuracy. The adaptive chaos synchronization algorithm combined with grid search avoids local minima even when the choice of initial values of the parameters is poor. Although we display results corresponding to one cycle for clear visualization, the prediction closely overlap the input data for the entire six-cycle data set

Adaptive chaos synchronization according to Huang [25] tracks the nonlinear chaotic signals with high accuracy even when the noise is increased or when data density is decreased. Figure 6a illustrates the tracking of cortisol model with noiseless data sampled at 1 min intervals. For which the method tracks the noiseless data with high accuracy. Figure 6b shows the system tracking when the input signal (“observed data”) includes 20% proportional noise.

Figure 6b, c illustrate what happens when the noise level is increased. This method is still capable of tracking the filtered data with high accuracy. Figure 6d shows the performance of this method when the noiseless system is sparsely sampled.

Table 3 shows that RMSE is low for chaos synchronization when compared to the classical least squares approach but increases with increase in noise while that for classical non-linear least squares regression RMSE remains almost constant because the synchronization signal adapts to accommodate the noise (Fig. 6b, c) so that the parameters can be estimated accurately. Also, as pointed out by Konnur [12] a system can possess multiple minima and, it is possible that the least squares method is converging to one of those local minima due to poor starting values or as pointed out by Jafari [31], poor initial conditions. However, with our fixed initial conditions, the adaptive chaos synchronization method converges to the global minimum.

#### Analyzing the effect of tuning parameters on convergence

**Fig. 7:**
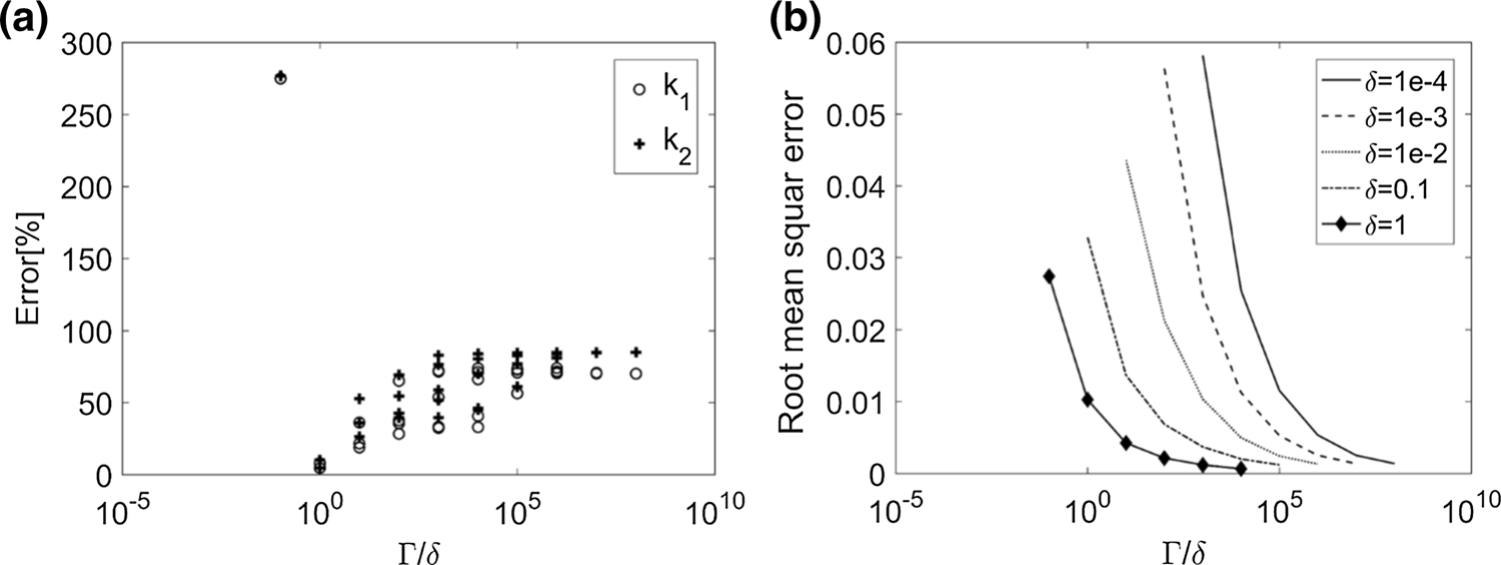
Variation of **a** percent error for *k*_*1*_ and *k*_*2*_ with the ratio of $\varGamma /\delta$**b** RMSE between the predicted and observed concentration with $\varGamma /\delta$. The root mean square error has units of concentration

For different combinations of $\varGamma$ and $\delta_{ }$ the final estimates of *k*_*1*_ and *k*_*2*_ were calculated and the Error [%] of estimated parameters and RMSE between observed and predicted concentration were plotted (see Fig. 7). The parameters $\delta_{ }$ and $\varGamma$ were observed to affect the rate of convergence and the accuracy with which predictions track the observed cortisol profile. The rate of convergence depends on the relative values of $\varGamma$ and $\delta_{ }$. It was observed that if $\delta > \varGamma$ the error [%] of estimation of *k*_*1*_ and *k*_*2*_ are very high (see Fig. 7). Since the observation data set has a finite duration, configurations for which the system converges to the true value of parameters in shorter time are expected to yield lower error[%] for the parameter estimate. From Fig. 7a it can be observed that when the ratio of $\varGamma /\delta$ is the same, the error[%] of estimation for *k*_*1*_ and *k*_*2*_ are similar.

In particular, note that clusters are formed for the cases that have similar ratio of $\varGamma /\delta$. From Fig. 7b one can observe that when the ratio of $\varGamma /\delta$ is increased the value of RMSE between the predicted and observed concentration decreases. Also, when $\varGamma /\delta = 1$ (Fig. 7a) the error[%] for parameter estimation were least. This information was used for selecting tuning parameters.

For grid search, we initially set $\varGamma /\delta$ to a high value. Once α and β were estimated using grid search we selected a lower value of $\varGamma /\delta = 1$ to accurately predict the value of *k*_*1*_ and *k*_*2*_ using adaptive chaos synchronization in the shortest possible time frame.

### Estimating linear and non-linear parameters

After comparing the performance of adaptive chaos synchronization, extended least squares and non-least squares regression for estimating linear parameters, our next step was to compare the performance of these methods while estimating the nonlinear parameters along with the linear parameters.

The previously discussed four scenarios were used to compare the performance of the combination of adaptive chaos synchronization and grid search with the combination of non-linear least squares regression and grid search and extended least squares with grid search to identify the nonlinear parameters.

#### Estimating non-linear parameters by combining adaptive chaos synchronization/non-linear least squares regression/extended least squares with grid search

Adaptive chaos synchronization according to Huang’s approach estimates only the linear parameters (*k*_*1*_ and *k*_*2*_). Our next step was to estimate those parameters which were nonlinear accurately. For a fair comparison between non-linear least squares regression, extended least squares and adaptive chaos synchronization, the nonlinear parameters were estimated using grid search in all three cases.

We performed a structural identifiability analysis using GenSSI 2.0 software based on a generating series method (e.g., Chis, O. et al., Bioinformatics 2011 Sep 15; 27(18):2610–2611). We described the delay in terms of the well-known Green’s function for a catenary of *m* first order transit compartments with individual transit time *ktr* expressed in terms of the overall delay *tau* by *ktr *=* tau/(m *+ *1)*.

We observed that the parameters we labeled in the manuscript as (α, β, *k*_*1*_,*k*_*2*_) are all locally structurally identifiable, however the circadian cycle phase is not identifiable separately from its amplitude.

Since the above quantities (α, β, *k*_*1*_,*k*_*2*_) are locally structurally identifiable for all *m*, they are also identifiable in the limit of crisp delay *tau* as *m *→ ∞.

Since we were able to uniquely identify the parameter combination (α, β, *k*_*1*_,*k*_*2*_), we decided to compare the performance of adaptive chaos synchronization with grid search to that of non-linear least squares with grid search and extended least squares with grid search while estimating these parameters with the phase parameter (t_f_) fixed based upon external knowledge of the nadir of the circadian cycle as may be obtained by experiment.

The parameter ‘n’ affects the oscillation of the cortisol secretion cycle and, its value is highly uncertain. From sensitivity analysis (see the next section) we observed that the concentration was moderately sensitive to changes in ‘n’. We thus decided to fix the value of ‘n’. Values of n > 5 were required to obtain a sufficiently sharp step for oscillations to occur. An excessively large value of ‘n’ resulted in system destabilization with increasing oscillations especially for n > 20. The fixed value n = 10 was chosen because the **dde23** solver was able to solve that model with high precision and accuracy.

**Fig. 8:**
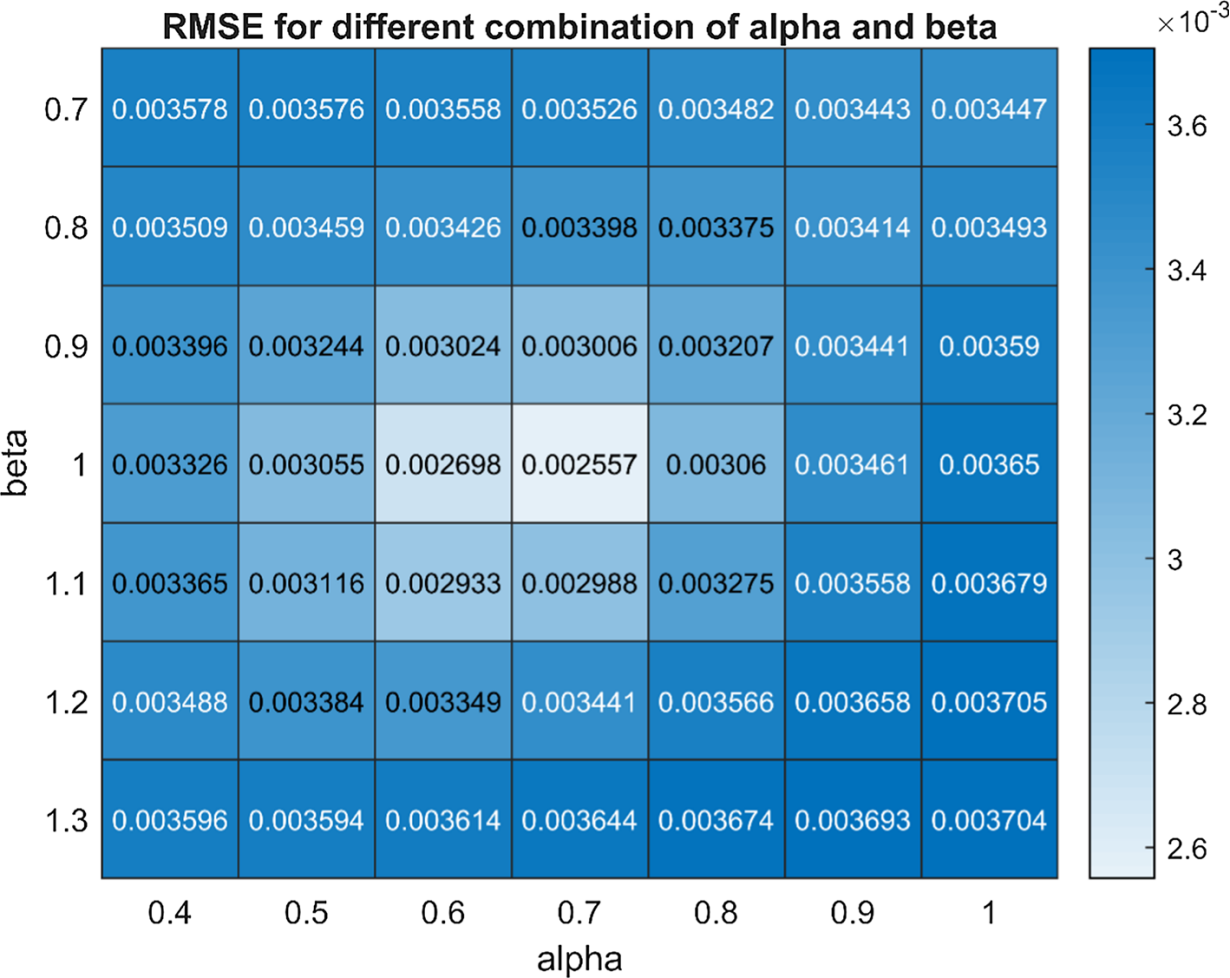
Variation in RMSE with change in alpha (α) and beta (β). From the plot, the grid for α and β can be clearly visualized. The method calculates the predicted concentration at each combination of α and β and compares it with the observed concentration to calculate the RMSE in units of concentration. The inset shows the location of the optimal (least) RMSE

Figure 8 illustrates the performance of the grid search. For visualization, the grid search is illustrated for optimization of parameters α and β with all other parameters set to their nominal values. For α = 0.7 and β = 1 an optimum configuration is obtained as the configuration with minimum RMSE. The value of the time delay d was fixed to 70 min which corresponds to about one cortisol secretion burst per hour in accordance with experimental observations and, the value of t_f_ was fixed to 250 [32].

#### Parameter estimation and signal tracking

Table 5 shows the result of parameter estimation using adaptive chaos synchronization/non-linear least squares regression/extended least squares combined with grid search.

**Table 5:** Estimation of parameters that enter in a linear fashion by chaos synchronization (CS), nonlinear least squares (NLS) and extended least squares (ELS)

Estimation method	Nominal	CS/grid search	NLS/grid search	ELS/grid search
Noiseless system with dense data^a^				
*k*_*1*_	0.0666	0.0690^d^	0.01^f^	0.0666^e^
*k*_*2*_	0.0333	0.0344^d^	0.01^f^	0.0333^e^
α	0.7	0.7^e^	0.8^e^	1^e^
β	1	1^e^	0.7^e^	0.7^e^
t_f_	250	Fixed	Fixed	Fixed
d	70	Fixed	Fixed	Fixed
n	10	Fixed	Fixed	Fixed
Noisy system^b^ with dense data^a^				
*k*_*1*_	0.0666	0.0629^d^	0.01^f^	0.0666^e^
*k*_*2*_	0.0333	0.0340^d^	0.01^f^	0.0333^e^
α	0.7	0.7^e^	0.7^e^	1^e^
β	1	1^e^	0.7^e^	0.7^e^
t_f_	250	Fixed	Fixed	Fixed
d	70	Fixed	Fixed	Fixed
n	10	Fixed	Fixed	Fixed
Noiseless system with sparse data^c^				
*k*_*1*_	0.0666	0.0615^d^	0.01^f^	0.0666^e^
*k*_*2*_	0.0333	0.0303^d^	0.01^f^	0.0333^e^
α	0.7	0.7^e^	1^e^	1^e^
β	1	1^e^	0.7^e^	0.7^e^
t_f_	250	Fixed	Fixed	Fixed
d	70	Fixed	Fixed	Fixed
n	10	Fixed	Fixed	Fixed

To estimate parameters for the noisy system we filter the data using the **wden** function provided by MATLAB (MathWorks: MA) version R2017a as input to the CS, NLS or ELS method

^a^Dense data signifies data sampled at 1 min intervals

^b^Noisy system signifies 20% proportional error

^c^Sparse data signifies data sampled at 45 min intervals

^d^Parameter estimated with adaptive chaos synchronization method

^e^Parameter estimated using grid search method

^f^Parameter estimated using least squares method (MATLAB **lsqcurvefit**)

**Fig. 9:**
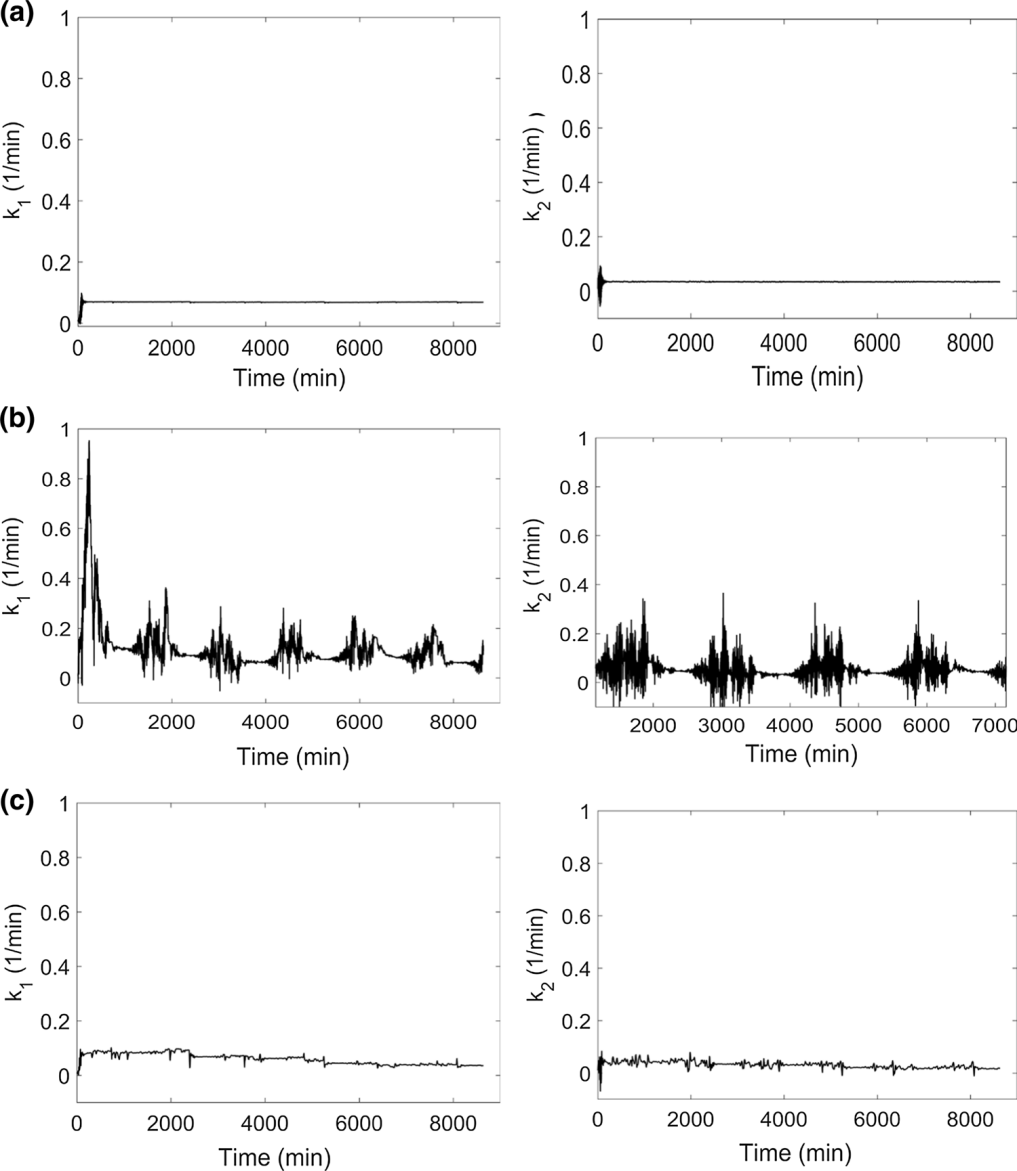
Estimation of cortisol model parameters for **a** noiseless data sampled at 1 min intervals, **b** data with with 20% proportional error sampled at 1 min intervals, **c** noiseless data sparsely sampled at 45 min intervals. The model was simulated for 8640 min (6 days) duration to observe the rate of parameter convergence. The noiseless case **a** resulted in parameter convergence closer to nominal values. The noisy **b** and sparsely sampled **c** cases resulted in fluctuation of the parameters about their nominal values, for which parameters were extracted by calculating the median of these fluctuating values. The noisy data were filtered by wavelet denoising. We densified the sparsely sampled data using the **pchip** function in MATLAB with output sampled at 5 min intervals

Figure 9a and Table 5 show that when the data are dense and noiseless, all the parameters converge close to their true value. When sparse and noisy data (Fig. 9b, c) drive parameter estimation, the estimated parameters fluctuate about their true values. The median was computed from these fluctuating values to extract the parameter value using the MATLAB **median** function with input as the output vector (of respective parameters) obtained from the output of the **dde23** solver.

For noisy and dense data, the parameter estimates were extracted as the median of the data values corresponding to the last cycle while for the sparse data, the parameter estimates were determined by the median of the entire output vector. For the sparsely sampled case, the **median** function required an increased number of data points to accurately estimate the median of the parameter value compared to the densely sampled case.

Combination of nonlinear least squares regression and grid search resulted in parameters converging to inaccurate values in all three cases (Table 5). The starting values of the parameters for the nonlinear least squares regression method are provided in Table 1 with final parameter values shown in Table 5.

The performance of systems tracking based on the combination of least squares and grid search is shown in supplementary section. The error is large when compared to the error obtained when we were estimating only the linear parameters and the system was being tracked with only nonlinear least squares regression (see Table 3).

**Fig. 10:**
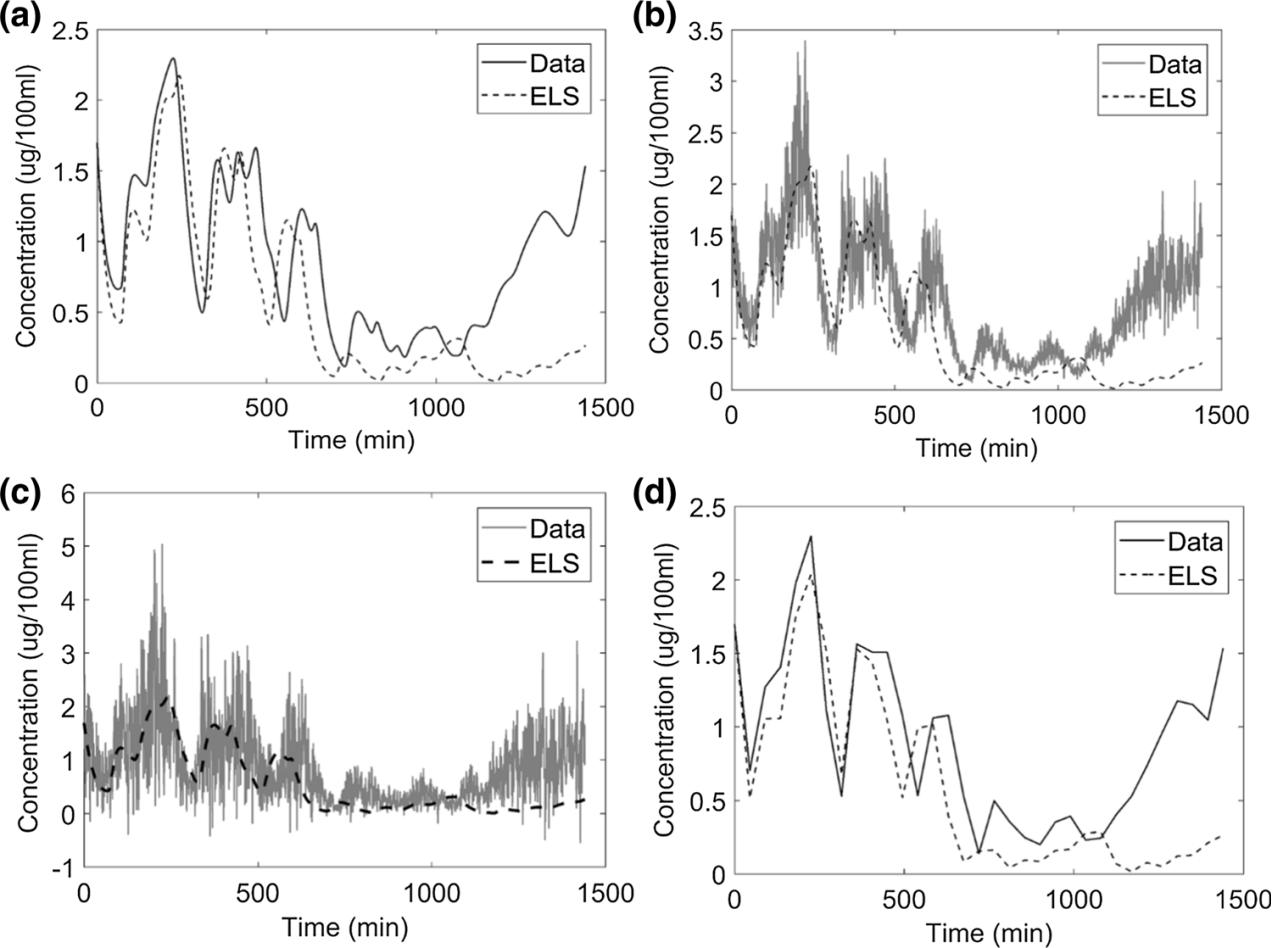
System tracking performance of the Extended Least Squares regression method: **a** noiseless data sampled at 1 min intervals, **b** data with 20% proportional error, **c** data with 50% proportional error, **d** noiseless data sparsely sampled at 45 min intervals. The data were filtered by wavelet denoising. ELS denotes predictions based on the ELS regression method. For clarity, we exhibit data corresponding to 1 cycle

Figure 10 shows the performance of system tracking based on the combination of extended least squares and grid search. The error is large when compared to the error obtained when we were estimating only the linear parameters (error was zero since the parameters converged to their true value). The error in this case is intermediate when compared to nonlinear least squares with grid search and chaos synchronization with grid search (see Table 3).

When estimating the system with the combination of chaos synchronization and grid search the grid search accurately estimated the nonlinear parameters α and β. Since nonlinear parameters were accurately estimated, we were able to determine the linear parameters (*k*_*1*_ and *k*_*2*_) with the same accuracy as when we estimated only the linear parameters using adaptive chaos synchronization. Hence, system tracking for the combination of grid search and chaos synchronization is the same as that shown in Fig. 6 and the error values are the same as those shown in Table 3.

It was not possible to estimate all parameters simultaneously using adaptive chaos synchronization according to Huang’s method alone since the parameters α and β appear in a nonlinear manner.

### Sensitivity analysis

We evaluated the parameter sensitivity of the predicted cortisol concentration.

**Fig. 11:**
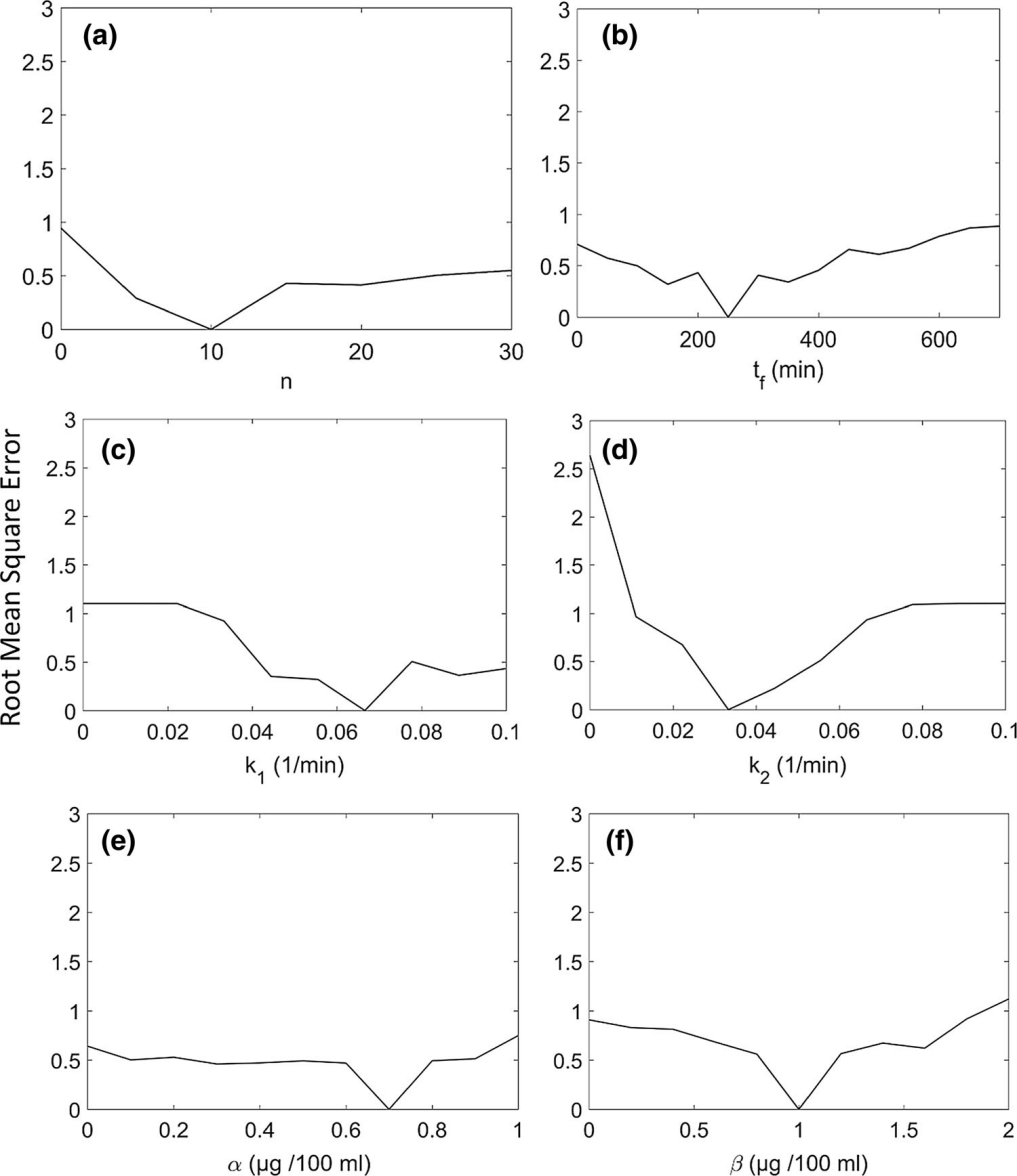
Sensitivity analysis for parameter (**a**) n, (**b**) *t*_*r*_, (**c**) *k*_*1*_, (**d**) k2, (**e**) α and (**f**) β. Increments of 0.01/min in either input or output cortisol rate constant significantly impacted the predicted concentrations. An increment of 100 min in phase had a relatively small effect on the predicted concentrations. Variation of parameters α, β and n has a moderate impact on predicted concentrations

Figure 11a demonstrates that when the exponent ‘n’ is varied from 1 to 20 in steps of 1 while fixing the values of the other parameters to their nominal value (Table 1), a minimum is obtained at n = 10. At this position, the error is close to zero. Any deviation from n = 10 increased the error value. Thus, n = 10 was considered the optimal value of ‘n’. Similarly, for parameter t_f_ the least error was obtained when value of t_f_ was set to 250.

From Fig. 11 it can be observed that the predicted concentration is highly sensitive to changes in input or output rate constant (*k*_*1*_ or *k*_*2*_), moderately sensitive to the constants α, β and fixed switch exponent ‘n’ and only slightly sensitive to changes in phase ‘t_f_’.

## Discussion

Parameter estimation and signal tracking for chaotic systems using a nonlinear least squares regression approach with grid search were considerably less accurate than adaptive chaos synchronization with grid search (see supplementary). Extended least squares method was found to be accurate when only linear parameters were being estimated but when both linear and nonlinear parameters were being estimated it was found to be less accurate than the adaptive chaos synchronization method. This illustrates the limitations of classical approaches for estimating non-linear physiological systems and is likely attributable to the multiple minima often found with nonlinear systems.

The adaptive chaos synchronization with grid search method appears to be more robust than non-linear least squares regression with grid search and extended least squares with grid search. Unlike classical approaches, adaptive chaos synchronization helps to avoid the tendency of the gradient-based optimization algorithms to converge to a local minimum. Parameter estimation and system tracking using this hybrid method with noiseless data with data sampled at 1-min intervals performed well and the parameters converged to about the nominal values provided in Table 1 (see Figs. 6, 9). In the presence of 20% or 50% proportional noise, this method was able to track the signals with high accuracy and, the estimated parameters (Table 5) are close to the nominal values (see Figs. 6, 9).

When the data were sparsely sampled (at 45-min intervals), the method was still able to track the system well and, the parameters converged close to their nominal values (Tables 1, 5, Fig. 9) by contrast with nonlinear least squares regression where we observed a substantial offset between the data and model predictions (see supplementary). For all the cases we analyzed, the adaptive chaos synchronization with grid search method converged to the global minimum and was robust to the selection of starting values and the nonlinear least squares regression with grid search and extended least squares with grid search most likely converged to a local minimum and thus failed to estimate the parameters accurately. In case of extended least squares for all cases of noise and data density, it was observed that the algorithm was not able to discriminate between parameters α and β, which may be due to convergence to local minima.

Parameter estimation required about six cortisol cycles to converge. It may be challenging to obtain samples for six cycles since this would require observing a subject for six consecutive days with regular sampling, although this is likely achievable via microdialysis.

We achieved similar results by replicating a single cycle six times and storing it in an array as input for parameter estimation via adaptive chaos synchronization. This is an approach that may be applied to all periodic systems. If a system is not periodic or the data are not sufficiently dense, we can achieve comparable results by recursive feedback i.e. feeding the output after a cycle as an input to the next cycle until the method converges with adequate accuracy.

It is also possible to densify the data using the **pchip (**Piecewise Continuous Hermite Interpolating Polynomial) function in MATLAB. This function requires three inputs, two of them are the predicted concentration and the corresponding time point and we can generate observations at the required time points by providing the required time points as the third input.

The proposed method was highly accurate for data sampled at intervals of up to 45 min. The percent error obtained for this case is less than 10% for each parameter (see Table 6). If the sampling interval exceeds about 45 min, the percent error is substantially increased. A plausible explanation is that there is a loss of geometric information as the shape of the cycle changes dramatically when the sampling interval is greatly increased. The parameters α and β affect the shape, thus affecting the estimates of *k*_*1*_ and *k*_*2*_.

**Table 6:** Percent error for parameter estimation using chaos synchronization and grid search

Dataset	Percent error in *k*_*1*_	Percent error in *k*_*2*_
Dense sample^a^-no noise	3.6	3.3
Dense sample^a^-20% proportional noise^b^	5.56	2.1
Sparse sample^c^-no noise	7.66	9.01

The remaining free parameters were estimated via grid search and they converged to the nominal values listed in Table 1

^a^Data sampled at 1 min intervals

^b^Data sampled at 1 min interval with 20% proportional error

^c^Noiseless data sampled at 45 min intervals

Grid search requires prior information regarding the range/order of actual parameter value to optimize computation. Iteratively grid search can be slow depending on the size of the grid. To speed up the process one can parallelize the nested for loops and pass a different set of parameter combinations to each core.

Nonlinear least squares regression may be able to track the chaotic system if the starting values are close to the true parameter values. When the starting value of *k*_*1*_ was changed to 0.03 and that of *k*_*2*_ was changed to 0.045, we observed that the least squares signal could track the model (see supplementary) with some offset and, this resulted in a decrease in the value of the RMSE although this value was still much greater than that obtained by the adaptive chaos synchronization method. For this set of starting values, the adaptive chaos synchronization method produced approximately the same result as when the starting values of *k*_*1*_ and *k*_*2*_ were set to 0.01 (see supplementary).

Jafari et al. [31] pointed out that for chaotic systems in general, the least squares metric is sensitive to initial conditions and may have bifurcations and intermittent windows typically associated with chaos. He shows the example of a logistic map for which the least squares metric has the global minimum in the wrong place.

Considering this possibility, we evaluated the effect of 0.1% change in the initial concentration value (see supplementary). We observed that when the initial concentration was changed to 1.7017 μg/100 ml there was a 52% increase in RMSE when the data was fitted for *k*_*1*_ and *k*_*2*_ alone using nonlinear least squares regression, approximately 5.7% increase in RMSE value when the data were fitted with chaos synchronization and 0% change in RMSE when the data were fitted with extended least squares. It is possible that the issue pointed by Jafari et al. [31] could be relevant to the poor performance of the nonlinear least squares regression method.

Applications of this approach are not restricted to chaotic pharmacologic systems but also apply to non-chaotic pharmacologic systems, reaction networks, secure message transmission using parameter modulation, aerospace, turbulence, meteorological and financial modeling among others [33, 34].

This approach may, in principle, be extended to the determination of models using machine learning methods that combine adaptive chaos synchronization with derivative-free optimization methods including genetic algorithms that encompass both the composite fitness function and a true multi-objective approach that describes outcomes, with applications such as oncology, psychiatry and cardiovascular disease.

## Conclusion

Our analysis shows that adaptive chaos synchronization avoids the tendency of a least squares gradient-based optimization method to converge to a local minimum. In combination with grid search, this method can effectively track trajectories and estimate parameters of the cortisol cyclic chaotic system. This method is robust against the effects of noise and changes in data sampling rate and hence may be of value for modeling non-linear physiological systems.

## Electronic supplementary material

Below is the link to the electronic supplementary material.

Supplementary material 1 (DOCX 2096 kb)

## References

[1] Van Rossum JM, de Bie JE (1991). Chaos and illusion. Trends Pharmacol Sci.

[2] Sheiner LB (1997). Learning versus confirming in clinical drug development. Clin Pharmacol Ther.

[3] Bies RR, Gastonguay MR, Schwartz SL (2008). Mathematics for understanding disease. Clin Pharmacol Ther.

[4] Danhof M (2016). Systems pharmacology—towards the modeling of network interactions. Eur J Pharm Sci: Off J Eur Fed Pharm Sci.

[5] Bakshi S, de Lange EC, van der Graaf PH, Danhof M, Peletier LA (2016). Understanding the behavior of systems pharmacology models using mathematical analysis of differential equations: prolactin modeling as a case study. CPT: Pharmacomet Syst Pharmacol.

[6] Dokoumetzidis A, Iliadis A, Macheras P (2001). Nonlinear dynamics and chaos theory: concepts and applications relevant to pharmacodynamics. Pharm Res.

[7] Gontar V (1997). Theoretical foundation for the discrete dynamics of physicochemical systems: chaos, self-organization, time and space in complex systems. Discret Dyn Nat Soc.

[8] Tallarida RJ (1990). On stability and control of ligand-receptor interactions according to the mass action law: a theoretical model of chaos. Drug Dev Res.

[9] Freeman KA, Tallarida RJ (1994). A quantitative study of dopamine control in the rat striatum. J Pharmacol Exp Ther.

[10] Hellman L, Nakada F, Curti J, Weitzman ED, Kream J, Roffwarg H, Ellman S, Fukushima DK, Gallagher TF (1970). Cortisol is secreted episodically by normal man. J Clin Endocrinol Metab.

[11] Kuznetsov VA, Makalkin IA, Taylor MA, Perelson AS (1994). Nonlinear dynamics of immunogenic tumors: parameter estimation and global bifurcation analysis. Bull Math Biol.

[12] Konnur R (2003). Synchronization-based approach for estimating all model parameters of chaotic systems. Phys Rev E.

[13] Pillai N, Craig M, Dokoumetzidis A, Schwartz SL, Bies R, Freedman I (2018). Chaos synchronization and Nelder–Mead search for parameter estimation in nonlinear pharmacological systems: estimating tumor antigenicity in a model of immunotherapy. Prog Biophys Mol Biol.

[14] Mackey MC, Glass L (1977). Oscillation and chaos in physiological control systems. Science.

[15] Murray JD (1993). Mathematical biology I. An introduction.

[16] Goodwin BC (1965). Oscillatory behavior in enzymatic control processes. Adv Enzym Regul.

[17] Leloup J-C, Goldbeter A (1998). A model for circadian rhythms in drosophila incorporating the formation of a complex between the PER and TIM proteins. J Biol Rhythm.

[18] Dokoumetzidis A, Iliadis A, Macheras P (2002). Nonlinear dynamics in clinical pharmacology: the paradigm of cortisol secretion and suppression. Br J Clin Pharmacol.

[19] Xiao Y, Xu W, Li X, Tang S (2009). The effect of noise on the complete synchronization of two bidirectionally coupled piecewise linear chaotic systems. Chaos.

[20] Zhou C, Lai CH (2000). Analysis of spurious synchronization with positive conditional Lyapunov exponents in computer simulations. Phys D.

[21] Shuai JW, Wong KW, Cheng LM (1997). Synchronization of spatiotemporal chaos with positive conditional Lyapunov exponents. Phys Rev E.

[22] Hastie T, Tibshirani R, Friedman J (2003) The elements of statistical learning: data mining, inference, and prediction. Springer, Berlin. 10.1007/b94608

[23] Peck CC, Beal SL, Sheiner LB, Nichols AI (1984). Extended least squares nonlinear regression: a possible solution to the “choice of weights” problem in analysis of individual pharmacokinetic data. J Pharmacokinet Biopharm.

[24] Boccaletti S, Kurths J, Osipov G, Valladares DL, Zhou CS (2002). The synchronization of chaotic systems. Phys Rep-Rev Sec Phys Lett.

[25] Huang D (2004). Synchronization-based estimation of all parameters of chaotic systems from time series. Phys Rev E.

[26] Lasalle JP (1960). The extent of asymptotic stability. Proc Natl Acad Sci USA.

[27] Lyapunov AM (1992). The general problem of the stability of motion.

[28] Pecora LM, Carroll TL (1990). Synchronization in chaotic systems. Phys Rev Lett.

[29] Huang D (2004). Synchronization-based estimation of all parameters of chaotic systems from time series. Phys Rev E: Stat, Nonlinear, Soft Matter Phys.

[30] Saltelli A, Ratto M, Andres T, Campolongo F, Cariboni J, Gatelli D, Saisana M, Tarantola S (2008). Global sensitivity analysis.

[31] Jafari S, Sprott JC, Pham V-T, Golpayegani SMRH, Jafari AH (2014). A new cost function for parameter estimation of chaotic systems using return maps as fingerprints. Int J Bifurc Chaos.

[32] Kraan GP, Dullaart RP, Pratt JJ, Wolthers BG, Drayer NM, De Bruin R (1998). The daily cortisol production reinvestigated in healthy men. The serum and urinary cortisol production rates are not significantly different. J Clin Endocrinol Metab.

[33] Revelli JA, Rodriguez MA, Wio HS (2010). Interplay between chaos and external noise in an extended system: improved forecasting due to intrinsic stochastic resonant phenomena. Int J Bifurc Chaos.

[34] Xie QX, Chen GR, Bollt EM (2002). Hybrid chaos synchronization and its application in information processing. Math Comput Model.

[35] Theesar SJS (2012). Adaptive synchronization in noise perturbed chaotic systems. Phys Scr.

