# Supplementary material for: Estimating parameters of nonlinear dynamic systems in pharmacology using chaos synchronization and grid search
Source: J Pharmacokinet Pharmacodyn. 2019 Mar 30;46(2):193–210. doi: 10.1007/s10928-019-09629-4 (PMC6491657; doi:10.1007/s10928-019-09629-4)
Supplement: Supplementary file 1 — Supplementary material 1 (DOCX 2096 kb) [file 10928_2019_9629_MOESM1_ESM.docx]

**Supplementary Information**


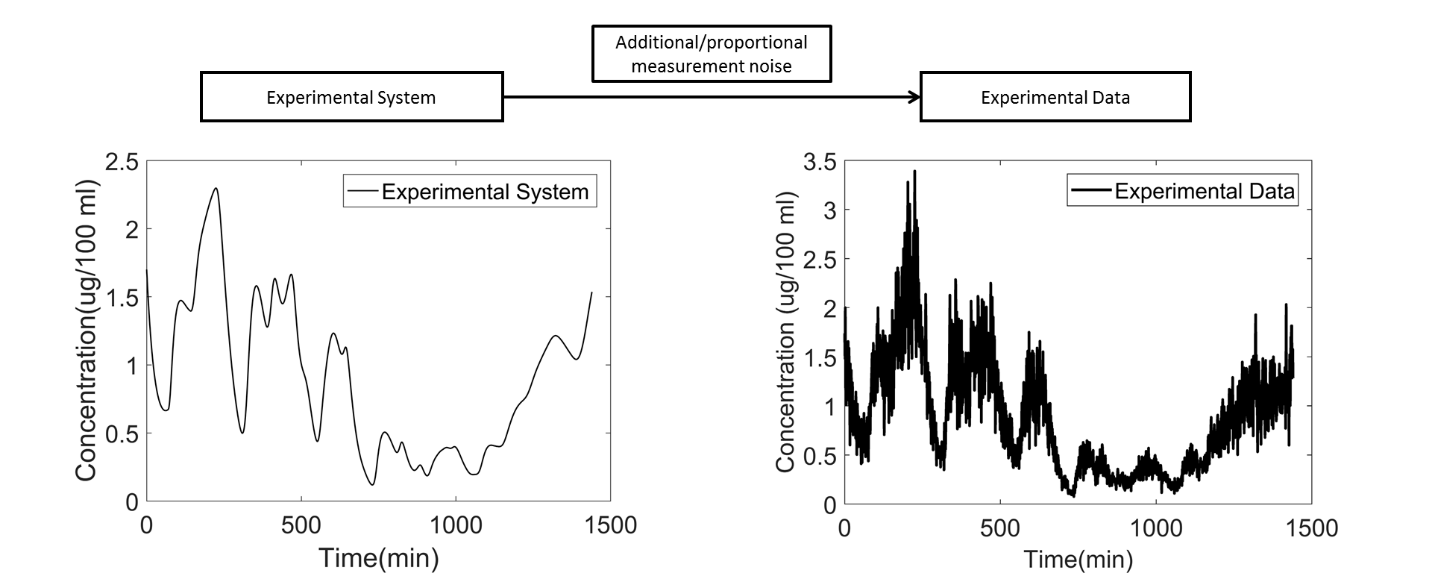


**Fig. S1** Noiseless simulation of the nominal cortisol model (Experimental System) and noisy simulation (Experimental Data).


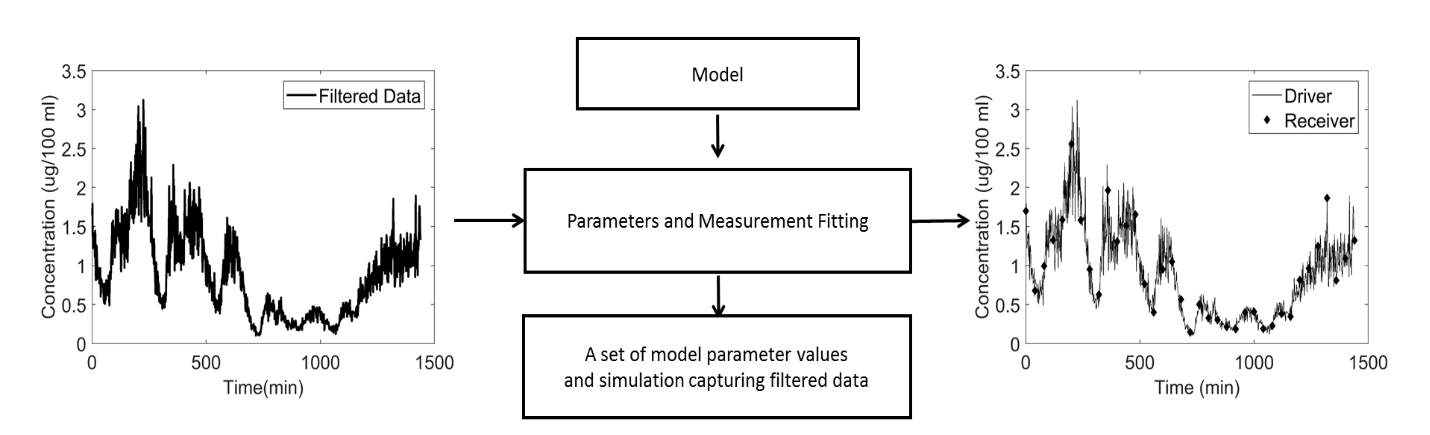


**Fig.S2** illustrates tracking the filtered signal obtained from noisy data generated by adding 20% proportional error to the experimental system for the nominal cortisol model. The data are sampled at 1 minute intervals. The filtered signal was obtained by wavelet denoising utilizing the **wden** function in MATLAB version 2017a.


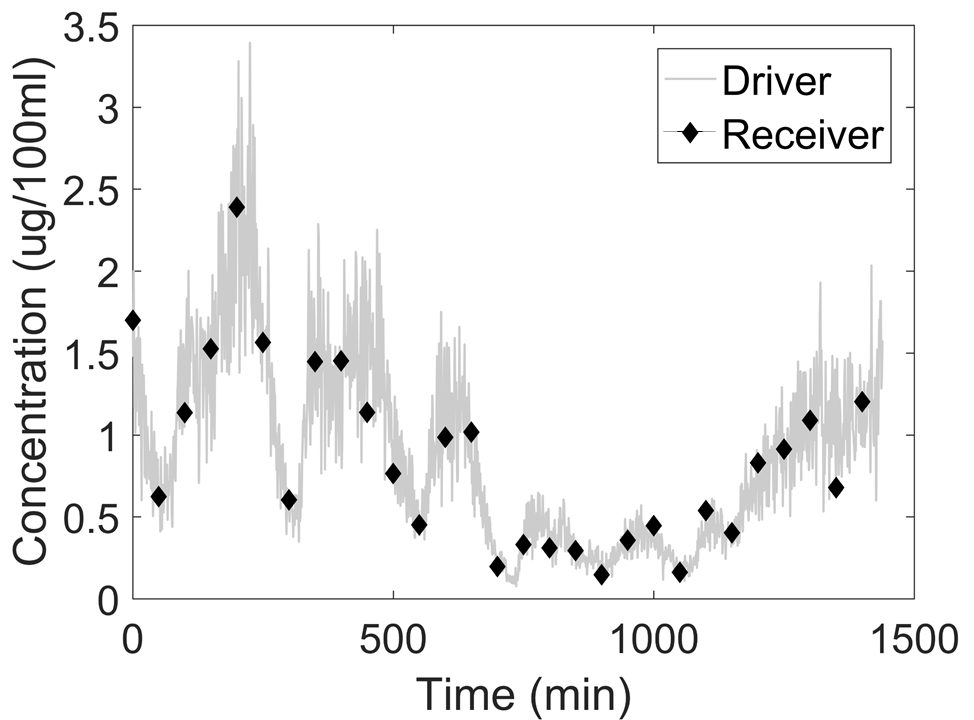


Fig.S3: Fitting an unfiltered dataset with 20% proportional error.using adaptive chaos synchronization


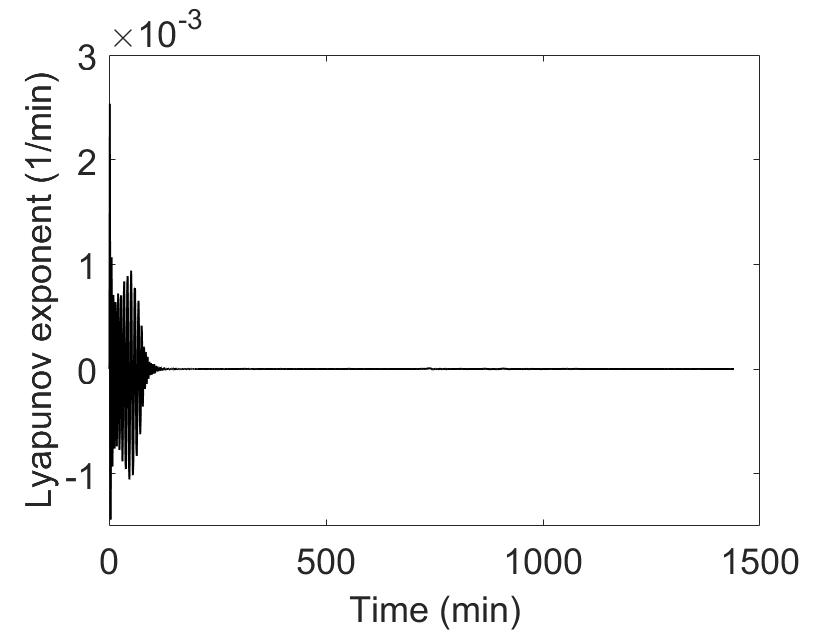


**Fig. S4** Leading Lyapunov exponent versus time for Γ=1 and δ=1 for a noiseless dense data set sampled at 1minute intervals. The leading Lyapunov exponent fluctuates and converges asymptotically to about zero.


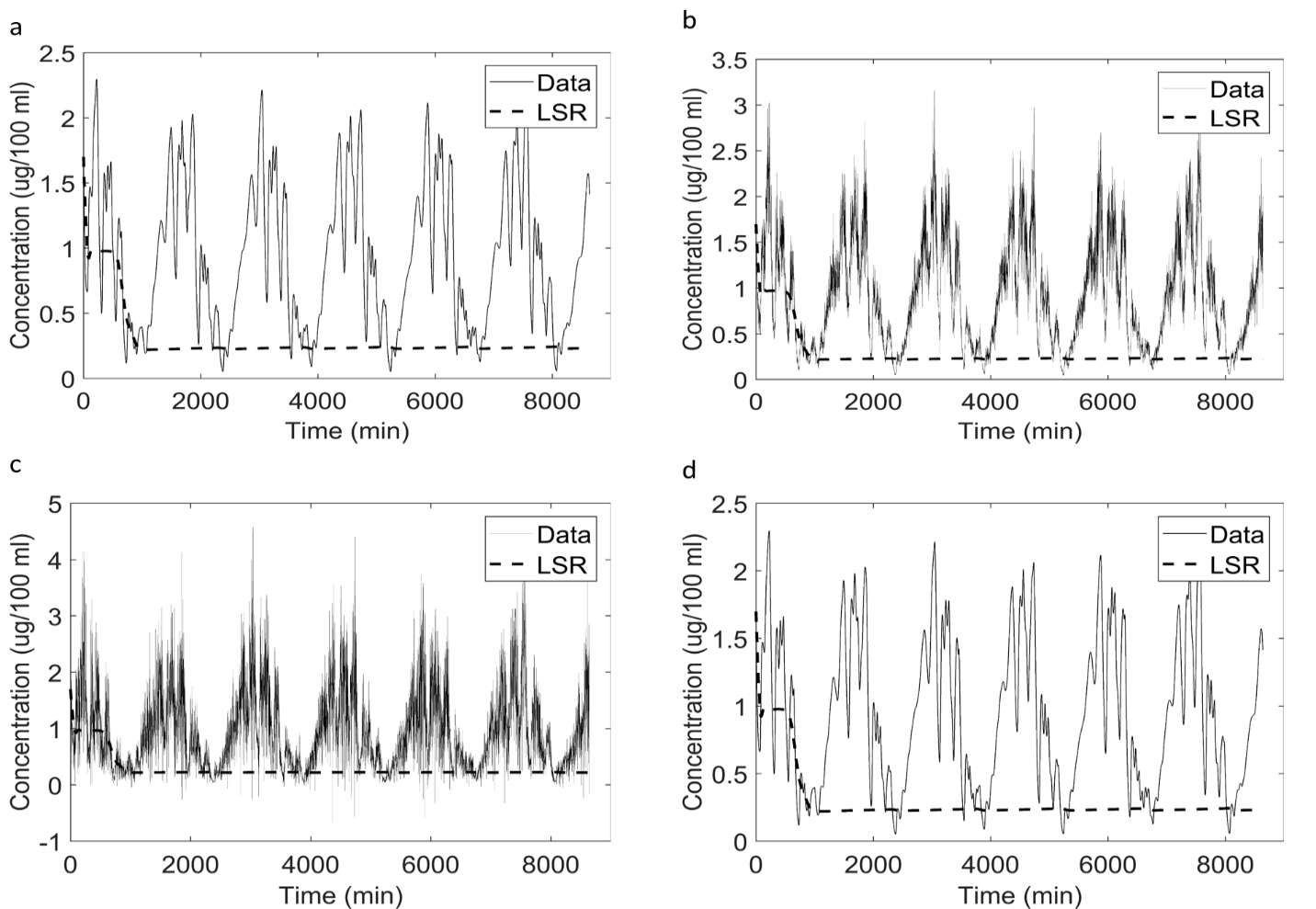


**Fig. S5** The system tracking performance of non-linear least squares regression: a) noiseless data sampled at 1 minute intervals; b) data with 20% proportional error sampled at 1 minute intervals; c) data with 50% proportional error sampled at 1 minute intervals ; d) noiseless sparse data sampled at 45 minute intervals. The filtering was performed using a **wden** filter. LSR represents the fit obtained using non-linear Least Squares Regression. The system converges to a local minimum.


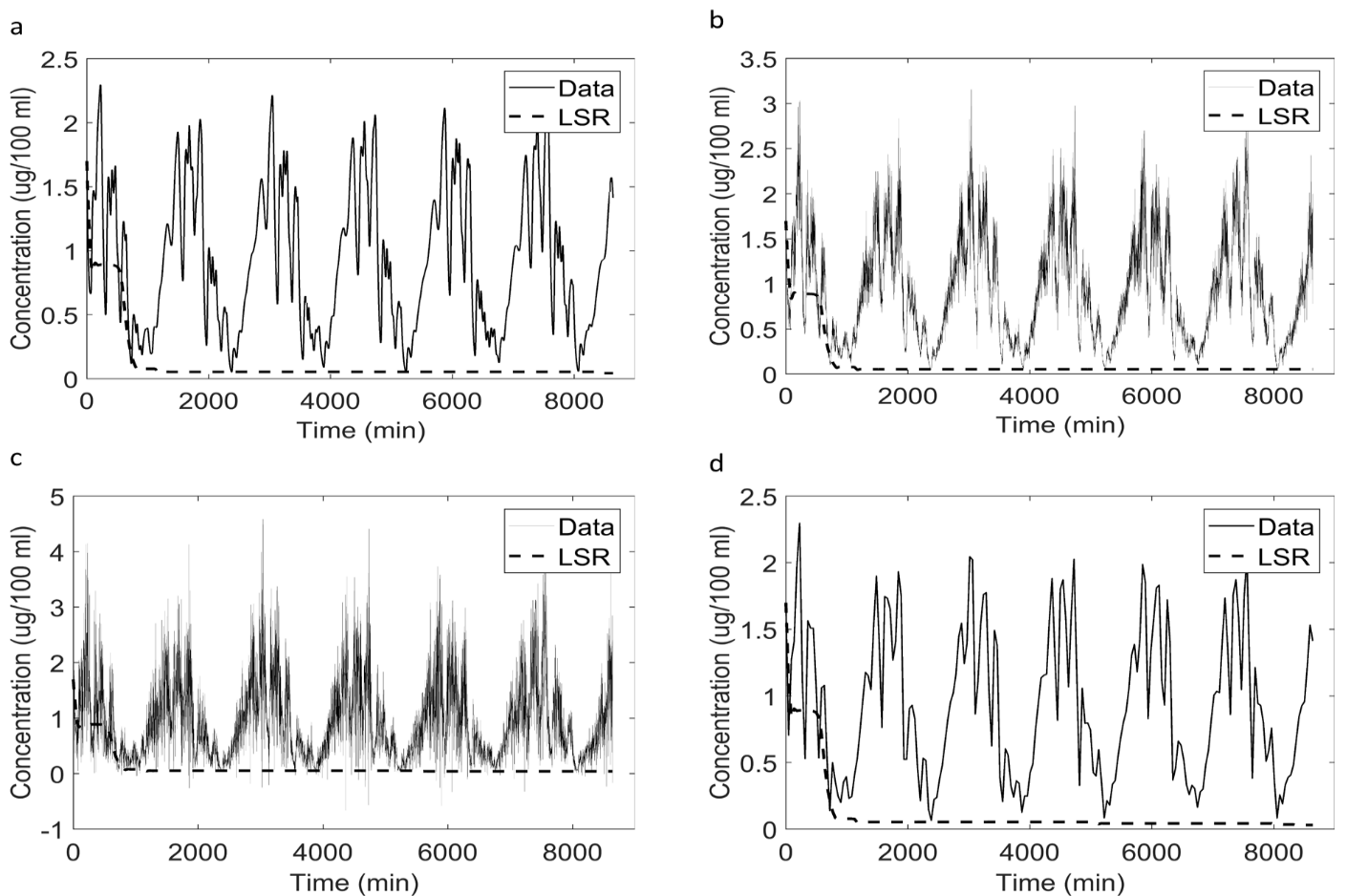


**Fig S6** Shows the performance of the combination of nonlinear least squares regression and grid search for system tracking: a) noiseless data sampled at one minute intervals, (b) noisy data with 20% proportional noise and with data measured at one minute intervals, c) data with high noise (with 20% proportional error) and with data measured at one minute intervals, d) noiseless data sparsely sampled at 45 minute intervals. The filtering was performed using a **wden** function in MATLAB version 2017a. LSR denotes the fit obtained using nonlinear least squares regression for which the system converges to a local minimum.


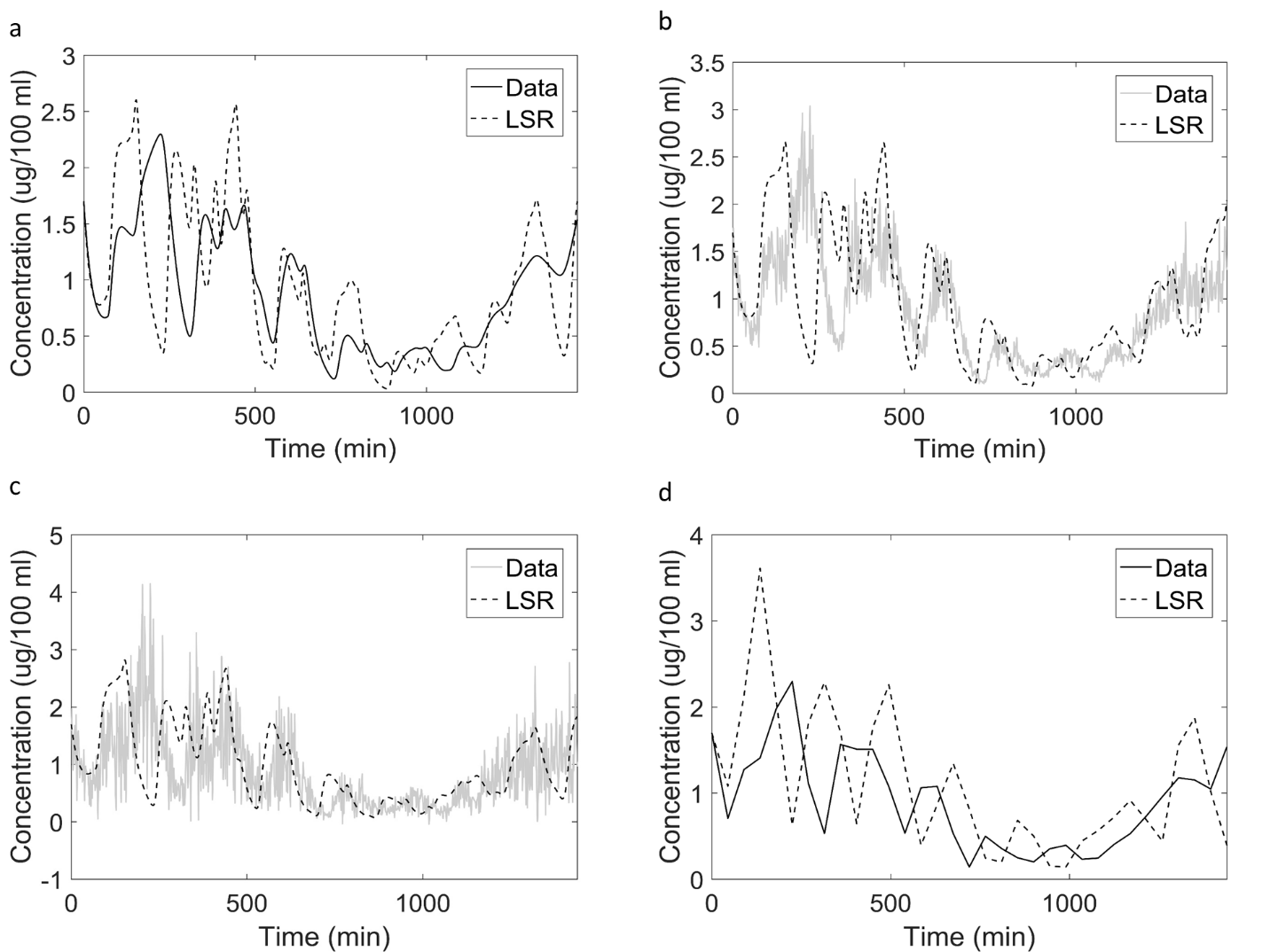


**Fig. S7** Shows the performance of the combination of nonlinear least squares regression and grid search for system tracking: a) noiseless data sampled at one minute intervals, (b) noisy data with 20% proportional noise and with data measured at one minute intervals, c) data with high noise (with 20% proportional error) and with data measured at one minute intervals, d) noiseless data sparsely sampled at 45 minute intervals. The filtering was performed using a **wden** function in MATLAB version 2017a. LSR denotes the fit obtained using nonlinear least squares regression. Starting values of k_1_ and k_2_ are 0.03 and 0.045 respectively. For clarity, we show data corresponding to one cycle.


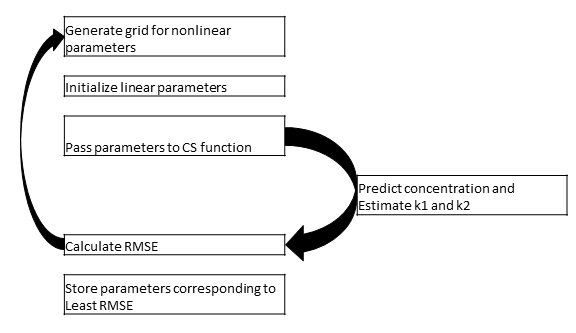


**Fig. S8** illustrates the algorithm. First, we create the grid for parameters α and β_._ Then we initialize parameters k_1_ and k_2_. Next, we pass these parameters to the chaos synchronization function which predicts the value of concentration at each time step and estimates the value of k_1_ and k_2._ We then compare the predicted and observed concentrations and calculate the Root Mean Square Error (RMSE). We repeat these steps until the entire grid has been searched. After this step we have obtained RMSE values corresponding to each parameter set. In the final step, we store the parameter values corresponding to combination that resulted in the least RMSE and these parameters correspond to the final estimates.

| **Data set** | **Nominal** | **CS/wden filter** | **CS/no filter** |
| --- | --- | --- | --- |
| **Noisy system (20% proportional error) with dense data (1 min sampling)** | | | |
| *k_1_* | 0.0666 | 0.0629 | 0.0692 |
| *k_2_* | 0.0333 | 0.0340 | 0.0362 |
| RMSE | 0 | 0.0131 | 0.0099 |

Table S1. Parameter estimates and RMSE value for data 20% proportional error with and without **wden** filter.

| **Estimation Method** | **Error-CS** | **Error-NLS** |
| --- | --- | --- |
| No noise, Dense data^1^ | 3.32e-04 | 0.4661 |
| Proportional error 0.2, Dense data^1^ | 0.0131 | 0.6155 |
| Proportional error 0.5, Dense data^1^ | 0.0162 | 0.6553 |
| No noise, Sparse data^2^ | 0.0019 | 0.9040 |

Table S2: RMSE between the predicted and observed concentration for various methods of estimation. The error is measured over one cortisol cycle. Units of error are $\mu$g /100ml. 1=data sampled at 1 minute intervals, 2:= data sampled at 45 minute intervals. Starting values of k_1_ and k_2_ are 0.03 and 0.045 respectively.

| **Estimation Method** | **Nominal** | **CS** | **NLS** |
| --- | --- | --- | --- |
| **Noiseless system with dense data^a^** | | |  |
| *k_1_* | 0.0666 | 0.0698 | 0.1210 |
| *k_2_* | 0.0333 | 0.0346 | 0.0422 |
| **Noisy system^b^ with dense data^a^** | |  |  |
| *k_1_* | 0.0666 | 0.0629 | 0.1187 |
| *k_2_* | 0.0333 | 0.0340 | 0.0405 |
| **Noiseless system with sparse data^c^** | | |  |
| *k_1_* | 0.0666 | 0.0651 | 0.1182 |
| *k_2_* | 0.0333 | 0.0319 | 0.0397 |

Table S3. Parameter estimation by adaptive chaos synchronization and nonlinear least squares regression for various cases. The data were filtered was performed by a **wden** filter. LSR represents the fit obtained using nonlinear least squares regression. Starting values of k_1_ and k_2_ are 0.03 and 0.045 respectively.

a) Dense data signifies data were sampled at 1 minute intervals.

b) Noisy system signifies data with 20% proportional error

c) Sparse data signifies data points were sampled at 45 minute intervals.

| **Initial cortisol concentration (μg/100 mL)** | **RMSE** | | |
| --- | --- | --- | --- |
|  | **CS** | **LS** | **ELS** |
| 1.7 | 3.5e-04 | 0.3226 | 0.5013 |
| 1.7017 | 3.7e-04 | 0.4919 | 0.5013 |

Table S4. RMSE for 0.1% change in initial concentration. Starting values of *k_1_* and *k_2_*  were fixed to their nominal value. The error was calculated over one cycle.

| **Data set** | **Nominal** | **CS/wden level 1** | **CS/wden-level 4** |
| --- | --- | --- | --- |
| **Noisy system^b^ with dense data^a^** | |  |  |
| *k_1_* | 0.0666 | 0.0629 | 0.0852 |
| *k_2_* | 0.0333 | 0.0340 | 0.0406 |
| RMSE | 0 | 0.0131 | 0.0094 |

Table S5. Parameter estimates and RMSE value for different level of wavelet denoising performed on data with 20% proportional error via the **wden** function in MATLAB version 2017a (see Fig.S3).

| **Parameter** | **Nominal** | **CS/20% additive error** | **Percent Error** |
| --- | --- | --- | --- |
| *k_1_* | 0.0666 | 0.0739 | 10.9% |
| *k_2_* | 0.0333 | 0.0336 | 0.9% |
| RMSE | 0 | 0.0054 |  |

Table S6. Parameter estimates and RMSE value for data with 20% additive error.
